# A Highly Water-Soluble C_60_-Oligo-Lysine Conjugate as a Type I and Type II Photosensitizer with Enhanced ROS Generation and Photocytotoxicity

**DOI:** 10.1021/acsphyschemau.5c00023

**Published:** 2025-05-27

**Authors:** Yue Ma, Lorenzo Persi, Kateryna A. Tolmachova, Maxim Yulikov, Miroslav Peterek, Stephan Handschin, Nicola Armaroli, Barbara Ventura, Yoko Yamakoshi

**Affiliations:** † Department of Chemistry and Applied Biosciences, 27219 ETH Zürich, Vladimir-Prelog-Weg 3, CH-8093 Zürich, Switzerland; ‡ ScopeM, ETH Zürich, Otto-Stern-Weg 3, CH-8093 Zürich, Switzerland; § Istituto per la Sintesi Organica e la Fotoreattività, Consiglio Nazionale delle Ricerche (ISOF-CNR), Via P. Gobetti 101, 40129 Bologna, Italy

**Keywords:** fullerene-peptide conjugates, reactive oxygen species, type I electron transfer, transient absorption spectroscopy, photocytotoxicity

## Abstract

C_60_ has been regarded as a suitable photosensitizer for photodynamic therapy due to its excitation in the phototherapeutic window (650–900 nm), high quantum yields of ^1^O_2_ generation, and low dark toxicity. However, the use of this molecule in biomedical applications has been limited by its high aggregation tendency in polar solvents (e.g., water), resulting in quenching of its excited states. In this study, a C_60_-peptide conjugate, C_60_-oligo-Lys, with a lower aggregation tendency was investigated by chemical, physical, and photophysical methods in comparison to a previously reported water-soluble C_60_-PEG conjugate. Photoinduced ^1^O_2_ generation was evaluated by both phosphorescence at 1274 nm and the electron spin resonance method in an aqueous solution, with comparison to the control C_60_-PEG, revealing the superior capacity of the C_60_-oligo-Lys conjugate. Importantly, the photoinduced type I electron transfer reaction is occurring in C60-oligo-Lys very efficiently, even in the absence of an e^–^ donor, presumably due to the partially unprotonated amines in the peptide, to form O_2_^•–^ and ^•^OH, which are generated in a further enhanced way by the addition of a physiological concentration of NADH. These species are more harmful to the target cells, including hypoxic tissues with limited oxygen concentration. Femtosecond transient absorption spectroscopy revealed different excited state dynamics for C_60_-oligo-Lys and C_60_-PEG at short time scales in water. By an in vitro cellular assay, significant cytotoxicity of C_60_-oligo-Lys was observed (IC_50_ < 1 μM) on HeLa cells under visible light irradiation (527, 630, and 660 nm), while very limited cytotoxicity was observed for C_60_-PEG (IC_50_ > 25 μM) under the same conditions. The strongly enhanced photocytotoxicity of C_60_-oligo-Lys can be ascribed to the higher generation of both type I and type II ROS in addition to the potential affinity of the positively charged oligo-Lys moiety for the negatively charged cell membrane. The C_60_-oligo-Lys conjugate reported in this study therefore shows high potential as a core photosensitizer for photodynamic therapy.

## Graphical abstract

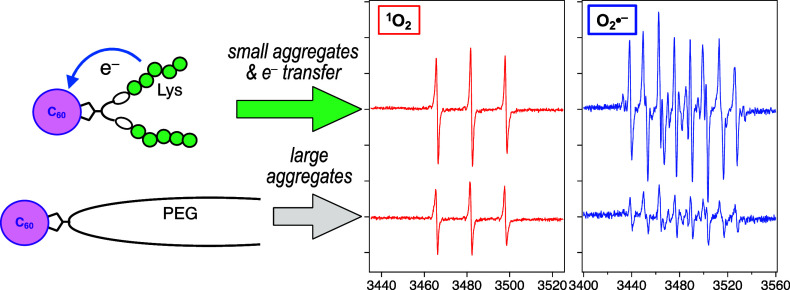

## Introduction

The excellent ability of fullerenes (C_60_ and C_70_) to generate reactive oxygen species (ROS) in high quantum yields^1–8^ renders them suitable as photosensitizers (PSs) for photodynamic therapy (PDT).^9–11^ Owing to their highly π-conjugated structures and small HOMO-LUMO gaps, fullerenes can be excited by visible light with relatively long wavelengths,^12,13^ which can better penetrate into tissues. However, the hydrophobic polyaromatic structures of fullerenes cause insolubility or aggregation of the molecules in water or water-miscible solvents, often hampering their biological applications. To overcome this obstacle, many efforts have been taken to develop water-soluble fullerene materials by (1) covalent chemical derivatization of the carbon sphere by introducing water-soluble moieties^14,15^ or (2) complexation of the hydrophobic fullerene cores with water-soluble carriers, e.g., cyclodextrins and calixarenes,^16,17^ vesicles,^18^ and polymers.^19^

Typically, covalently functionalized C_60_ derivatives form aggregates or clusters in water, in which the hydrophobic C_60_ core is located inside and the water-soluble moieties outside, potentially affecting the physicochemical properties of the molecules. Indeed, these aggregation phenomena often cause a faster deactivation of the fullerene triplet state as compared to organic solvents, where they are fully solubilized.^13,20,21^ Formation of such C_60_ aggregates can be disfavored by complexation with a surfactant or water-soluble host molecules, leading to the formation of long-lived triplets, detected by *T_1_–*T_*n*_ absorption.^22^ It is, therefore, apparent that achieving highly dispersed C_60_ derivatives is important for their function as PSs in biological environments. So far, only few studies have been reported on the effect of water-soluble moieties, covalently connected to the hydrophobic fullerene core, on ROS generation.^10^ It remains unclear how such hydrophilic anchors affect the excited state dynamics of the C_60_ core and, therefore, the generation of ROS in aqueous solutions.

Previously, we have reported several water-soluble C_60_ conjugates bearing non-ionic water-soluble polymer chains such as polyethylene glycol (PEG)^23^ and poly­(vinylpyrrolidone) (PVP).^24^ While these C_60_ conjugates are highly soluble in water and generated significant amounts of ROS under visible light irradiation,^7^ no significant photocytotoxicity was observed by in vitro cellular tests.^25^ Recently, to develop more biocompatible PSs, we synthesized three types of C_60_-peptide conjugates (oligo-Lys, oligo-Arg, and oligo-Glu) by connecting the C_60_ core with hydrophilic oligopeptides^26^ using a convenient solid-phase synthesis starting from a Prato derivative **3** (Figure 1), as a versatile and convenient starting material.^27^ Out of the three conjugates, only C_60_-oligo-Lys **1** (Figure 1) was found to be highly soluble in a neutral buffer. In this study, the photophysical properties and visible light-induced ROS generation of C_60_-oligo-Lys **1** are fully accounted for, in connection to its aggregation behavior in water, by means of absorption/emission spectroscopy; ^1^O_2_ phosphorescence detection; electron spin resonance (ESR) spin-trapping detection of ^1^O_2_, O_2_^•–^, and ^•^OH; and femtosecond transition absorption spectroscopy. The study has been conducted with reference to C_60_-PEG conjugate **2** (Figure 1), bearing a non-ionic water-soluble moiety and with similar molecular weight, as a control. Clear differences in the fate of the photoexcited fullerene core between the two water-soluble C_60_ conjugates **1** and **2** are reported, revealing the superior properties of C_60_-oligo-Lys **1** in ROS generation and photocytotoxicity and thereby demonstrating its potential as a PDT–PS agent.

**Figure 1:**
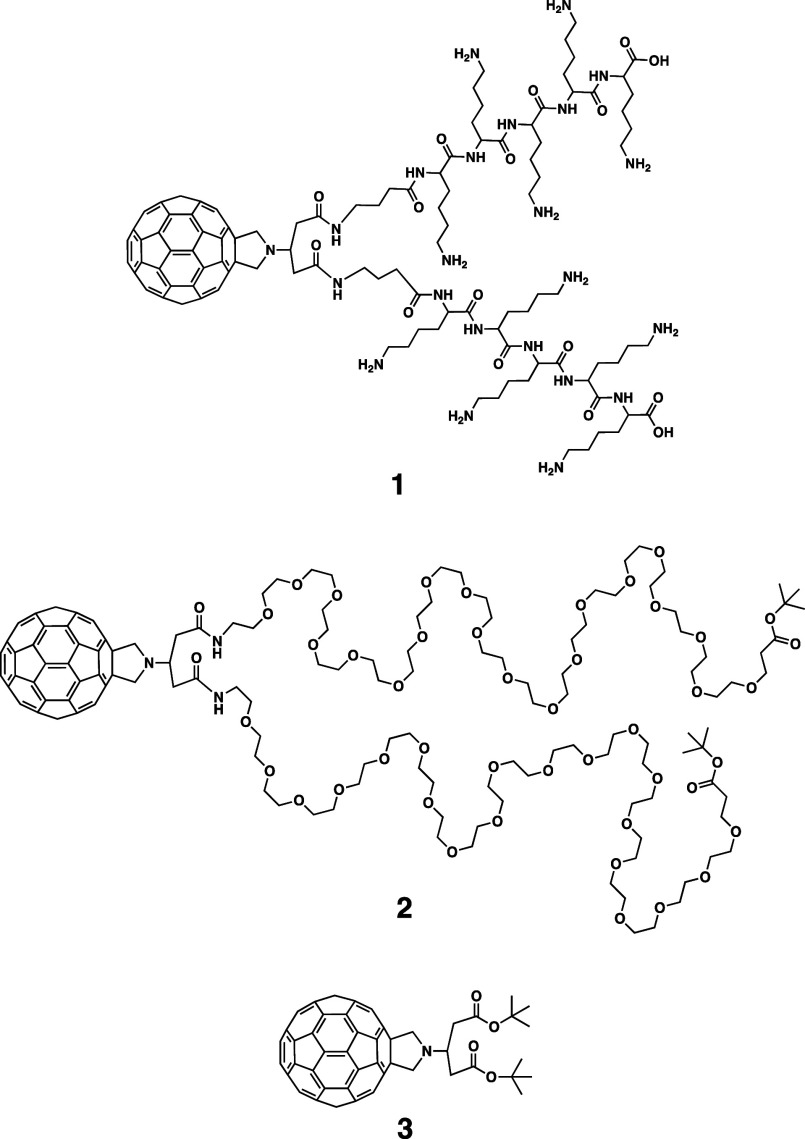
Structures of C_60_-oligo-Lys conjugate **1**, C_60_-PEG conjugate **2**, and fulleropyrrolidine mono-adduct **3**.

## Experimental Section

### Syntheses of C_60_-Oligo-Lys **1** and C_60_-PEG **2**

Fulleropyrrolidine **3** was synthesized based on the previously reported method by the Prato reaction,^27^ and deprotected to form C_60_-bis-carboxylic acid. This C_60_-bis-carboxylic acid was coupled to the terminal amine of the oligo-peptide (GABA-(Lys)_5_) on the resin using HBTU and DIPEA. Deprotection of the Lys residue and peptide cleavage from the resin were performed by TFA, TIPS, and water.^26^ C_60_-PEG **2** was synthesized by the coupling of C_60_-bis-carboxylic acid and amine-PEG using HBTU and DIPEA.^23^ Compounds **1** and **2** were purified by a reversed-phase HPLC.

### Absorption and Emission Measurements

Aqueous solutions of **1** and **2** were prepared in tridistilled Milli-Q water (adjusted to pH 7.0). Absorption spectra were recorded with a PerkinElmer Lambda 950 UV–Vis–NIR spectrophotometer (PerkinElmer, Inc.) in 1 cm quartz cuvettes, both with and without the use of a 100 mm integrating sphere. Emission spectra were collected in a right-angle setup with both an FLS920 spectrofluorimeter (Edinburgh Instruments Ltd.) equipped with a Peltier-cooled R928 PMT (280–850 nm, Hamamatsu Photonics) and an Edinburgh FLS920 fluorimeter equipped with a Hamamatsu R5509-72 InP/InGaAs photomultiplier tube supercooled at 193 K in a liquid nitrogen cooled housing and a TM300 emission monochromator with a NIR grating blazed at 1000 nm (300–1700 nm). The spectra have been corrected for the wavelength-dependent phototube response. Fluorescence quantum yields have been determined with reference to C_60_ (Sigma-Aldrich) in aerated toluene (ϕ_fl_ = 2.2 × 10^–4^)^28^ upon excitation at 340 nm. Fluorescence lifetimes were measured with an IBH Time Correlated Single Photon Counting apparatus with nano light-emitting diode (LED) excitation at 331 nm. The analysis of the luminescence decay profiles against time was accomplished with the DAS6 Decay Analysis Software provided by the manufacturer. The estimated error on molar absorption coefficients, luminescence lifetimes, and quantum yields is 10%.

### DLS Measurements

DLS measurements were performed on a Malvern Nano-ZetaSizer (Malvern Instruments Ltd.) equipped with a 5 mW HeNe laser (wavelength: 632.8 nm) and a digital logarithmic correlator.

### CryoTEM Imaging

An aliquot of **1** or **2** solution (1 mM) in Milli-Q water was added onto lacey carbon-coated copper grids (Electron Microscopy Sciences) or on holey carbon grids coated with thin carbon (R2/2 + 2 nmC, Quantifoil Micro Tools GmbH), which were previously negatively glow-discharged (Emitech K100X). Excess of sample was blotted away for two or two and a half seconds (for Quantifoil grids) using a Vitrobot Mark IV (Thermo Fisher Scientific) with the environmental chamber set to 22 °C and 100% humidity, and the grids were plunge-frozen in a mixture of liquid ethane/propane (continuously cooled by liquid nitrogen). The cryo-EM grids were loaded into a Titan Krios microscope operating at 300 kV (Thermo Fisher Scientific), equipped with a Gatan Quantum-LS Energy Filter and a Gatan K2 Summit direct electron detector (Gatan Inc.). The samples were imaged in the EFTEM mode using the Thermo Fisher Scientific EPU software (215,000× magnification, approximately 60 *e* ^–^/*A*2 total electron dose, K2 in linear mode) with a defocus range of −2 to −4 μm. Resulting micrographs were saved in a .tiff format using the DigitalMicrograph Software from Gatan Microscopy Suite.

### Singlet Oxygen Quantum Yield Determination by a Luminescence Method

D_2_O was purchased from VWR Chemicals and TPPS_4_ from Sigma-Aldrich, and they were used as received. Singlet oxygen production quantum yields of **1** and **2** in D_2_O^29^ were measured with reference to 5,10,15,20-tetrakis­(4-sulfonatophenyl)-porphyrin (TPPS_4_) (ϕ_Δ_ = 0.64),^30^ by comparing the intensity of singlet oxygen phosphorescence spectra, recorded with a NIR fluorimeter described above, from optically matched solutions. Excitation at 325 nm was performed with a HeCd laser (Kimmon Koha Co., Ltd.). To obtain oxygen-saturation conditions, the D_2_O solutions of the compounds were bubbled with pure oxygen for 5 min in custom gastight fluorescence cells.

### Detection of Singlet Oxygen (^1^O_2_) by ESR with Spin-Trapping Reagents

ESR measurements were carried out on a Bruker spectrometer (Bruker BioSpin, GmbH) equipped with a microwave bridge X-band EPR. An aliquot (35 μL) of each aqueous solution of **1** or **2** (40 μM) and 4-oxo-TEMP (spin-trapping agent, 80 mM) in an oxygen-saturated phosphate buffer (pH 7.0, 60 mM) was subjected to the light irradiation by a green LED (527 nm, 93 lm W^–1^, Osram Oslon SSL 150, Lumitronix LED-Technik GmbH) in a glass capillary (50 μL micropipette, Blaubrand intraMark), which was subsequently placed in an ESR tube (Φ4 mm × 250 mm, Wilmad) for the measurement under the following conditions: temperature 296 K; microwave frequency 10.03 GHz; microwave power 10 mW; receiver gain 5.0 × 10^4^; modulation amplitude 1.00 G; modulation frequency 100 kHz; sweep time 83.89 s; and scan times 10 times. Double integration of ESR spectra was performed on the WiNEPR processing program (Bruker BioSpin, GmbH).

### Detection of Superoxide Radical Anion (O_2_^•–^) by ESR with Spin-Trapping Reagents

Measurements were carried out in the same manner as ^1^O_2_ detection but using aqueous solutions of **1** or **2** (40 μM), with 5-diethoxyphosphoryl-5-methyl-1-pyrroline *N*-oxide (DEPMPO) (spin-trapping agent, 113 mM), NADH (electron donor, 0 or 10 mM), DETAPAC (chelator for Fe­(II), 1 mM), and l-histidine (^1^O_2_ quencher, 0 or 10 mM) in a 60 mM phosphate buffer.

### Detection of Hydroxyl Radical (^•^OH) by ESR with Spin-Trapping Reagents

Measurements were carried out in the same manner as ^1^O_2_ detection but using aqueous solutions of **1** or **2** (40 μM), with 5,5-dimethyl-1-pyrroline *N*-oxide (DMPO) (spin-trapping agent, 145 mM), NADH (electron donor, 0 or 10 mM), and Fe­(II)-DETAPAC (40 μM) in a 60 mM phosphate buffer.

### Transient Absorption Spectroscopy

Pump–probe transient absorption measurements were performed by means of a HELIOS (HE-VIS-NIR) (Ultrafast Systems) femtosecond transient absorption spectrometer by using, as an excitation source, a Solstice-F-1K-230 V laser system (Newport Spectra Physics), combined with a TOPAS Prime (TPR-TOPAS-F) (Light Conversion) optical parametric amplifier (pulse width: 100 fs, 1 kHz repetition rate, and selected output wavelength: 320 nm). The overall temporal resolution of the system is 300 fs. Air-equilibrated solutions in 0.2 cm optical path cells were analyzed under continuous stirring. The pump energy on the sample was 4 μJ/pulse. Surface Xplorer V4.5 software from Ultrafast Systems was used for data acquisition and analysis. The three-dimensional data surfaces were corrected for the chirp of the probe pulse prior to the analysis. Lifetimes were taken as the average of values derived from the fitting of several decays in selected ranges. Errors on lifetimes were estimated as the errors reported by the fitting software for each lifetime.

### Photocytotoxicity

Hela cells in log phase were seeded on in 96-well microtiter plates with a density of ca. 1 × 10^4^ per well and incubated in Dulbecco’s modified Eagle’s medium (DMEM) at 37 °C for 24 h with 5% CO_2_. The medium was removed from each well and replaced with a solution of **2** or **3** in DMEM (100 μL each) with varied concentration, and the cells were incubated for 24 h in the dark. The solution in each well was then removed, and the cells were washed with PBS(−) and replaced with DMEM (without phenol red) before photoirradiation. Photoirradiation was performed using Lumidox II 90-well LED Arrays (Analytical Sales and Services, Inc.) with LED lights with a maximum wavelength at 527, 630, or 660 nm. Cell viability was measured by a standard MTT method.

## Results and Discussion

### Absorption and Emission Properties of Conjugates **1** and **2**

C_60_-oligo-Lys conjugate **1** and C_60_-PEG conjugate **2** were synthesized from a fulleropyrrolidine derivative **1** based on our previously reported procedures^23,26^ and purified by reversed-phase HPLC. Upon addition of Milli-Q water (pH = 7.0), C_60_-oligo-Lys **1** and C_60_-PEG **2** afford transparent brownish solutions, suggesting that these two compounds are fully soluble in water.

Absorption spectra of **1** and **2** were recorded in Milli-Q water (pH = 7.0) (Figure 2a). The absorption spectrum of **1** exhibits typical absorption bands of the fulleropyrrolidine moiety (253, 320, 430, and 700 nm, Figure 2a, black lines),^22,31^ while C_60_-PEG **2** shows a broader and less resolved spectrum (Figure 2a, red lines), similarly to what reported for fulleropyrrolidine dendrimers with large appended branches.^32^ Measurements performed using an integrating sphere (Figure 2a, solid lines) provide evidence that scattering effects are more important for **2** than for **1**, indicating the formation of aggregates in the solution of the C_60_-PEG conjugate **2**.

**Figure 2:**
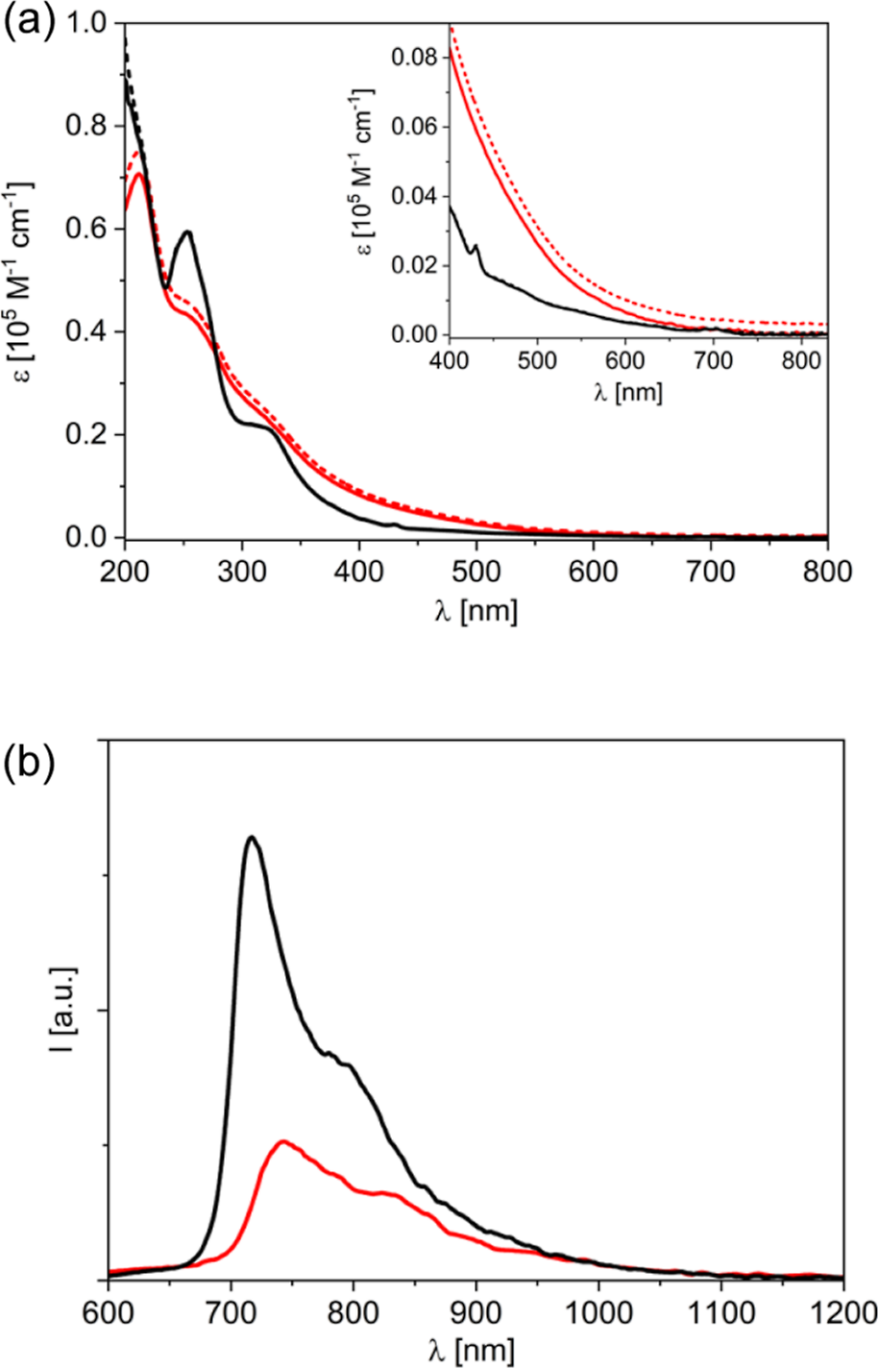
(a) Absorption spectra of C_60_-oligo-Lys **1** (black) and C_60_-PEG **2** (red) in Milli-Q water (pH = 7.0). Dashed lines: measurements in a conventional spectrophotometer setup; solid lines: measurements with the use of an integrating sphere. Inset: amplification of the 400–830 nm region. (b) Emission spectra from isoabsorbing solutions of **1** (black) and **2** (red) in Milli-Q water (pH = 7.0) with excitation at 340 nm (*A*_340_ = 0.13).

The emission spectra of **1** and **2** in water are shown in Figure 2b with the relative data collected in Table 1. Conjugate **1** displays the typical properties of fulleropyrrolidine compounds, in terms of spectral features, emission quantum yield, and lifetime.^33^ Conversely, **2** shows red-shifted fluorescence with a lower quantum yield and a multiexponential decay dominated by a short lifetime (ca. 100 ps, Table 1). This suggests that the C_60_-PEG derivative **2** is involved in aggregation phenomena causing quenching of the singlet excited state, while C_60_-oligo-Lys **1** behaves as a single molecule in aqueous solutions.

**Table 1:** Emission Parameters for **1** and **2** in Milli-Q Water (pH = 7.0)

	λ_max_ [nm]^a^	ϕ_fl_ ^b^	τ [ns]^c^
**1**	718, 790 sh	6.0 × 10^–4^	1.32
**2**	744, 830 sh	2.8 × 10^–4^	0.10 (86%),^d^ 0.66 (7%); 1.30 (7%)

a Emission maxima from corrected spectra.

b Fluorescence quantum yields measured with reference to C_60_ in aerated toluene (ϕ_fl_ = 2.2 × 10^–4^).^28^

c Excited state lifetimes, excitation at 331 nm (in brackets: fractional intensities).

d At the limit of the resolution of the single photon counting apparatus.

### Aggregation Properties of C_60_ Conjugates **1** and **2** by DLS and CryoTEM

Since the absorption and emission data described above indicate possible differences in the aggregation behavior of C_60_-oligo-Lys **1** and C_60_-PEG **2**, each aqueous solution was subjected to DLS measurements to investigate the aggregation phenomena. As shown in Figure 3a (top), freshly prepared solutions of **1** and **2** (1 mM in milli-Q water) reveal a single distribution of hydrodynamic diameters with a mean of <10 nm. When these aqueous solutions of **1** and **2** are kept at room temperature for 1, 2, and 30 days, clear changes are observed in the DLS diagram (Figure 3a). In the solution of **2** (dotted lines, Figure 3a), a small peak with a mean of >10 nm appears after 1 day, which is increased after 2 days, while no significant changes are observed in the solution of **1** (solid lines) within this time scale (Figure 3a). After 30 days, conjugate **2** exhibits larger aggregate formation (with a mean diameter of >100 nm) (Figure 3a, bottom, dotted line), while **1** forms relatively smaller aggregates with a mean of ca. 20 nm (Figure 3a, bottom, solid line).

**Figure 3:**
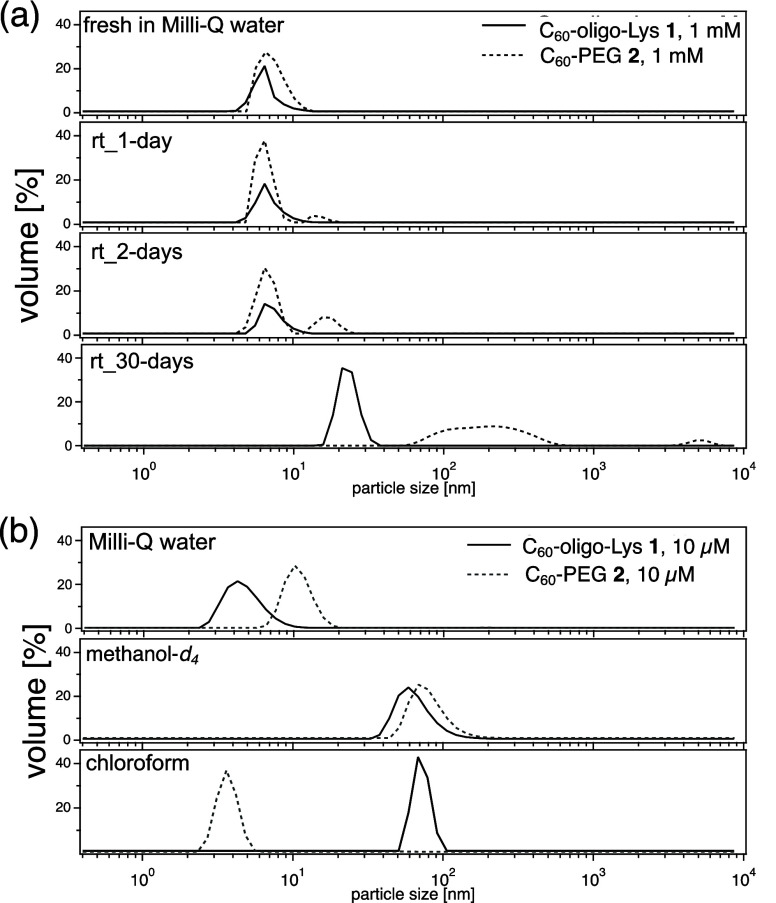
(a) Time-dependent DLS diagram of C_60_-oligo-Lys **1** and C_60_-PEG **2** in Milli-Q water (1.0 mM, at 25 °C, samples were measured as freshly prepared and 1, 2, and 30 days after). (b) Solvent-dependent DLS diagram of freshly prepared solutions of C_60_-oligo-Lys **1** and C_60_-PEG **2** (10 μM) in Milli-Q water, methanol-*d*_4_, and chloroform.

In our previous report, the formation of micelle-type morphologies was detected in aqueous solutions of C_60_-PEG molecules (>0.1 mM) by means of surface tension measurements at different concentrations.^23^ Furthermore, these C_60_-PEG conjugates formed larger aggregates at high temperature in a reversible manner, presumably due to the *gauche-*to-*anti* conformational changes of the PEG chains^34^ causing their dehydration. In the present study, it was expected that both C_60_ conjugates **1** and **2** formed some type of micelle due to their amphiphilic properties. The reduced aggregation tendency of **1** in water (Figure 3a) can be explained by the increased branching structure in the oligo-Lys moiety. This presumably hinders the formation of micelles with well-packed anchors, which is possible in the case of C_60_-PEG **2**. Furthermore, the lysine residues in the oligo-Lys anchors in **1**, which are at least partially protonated at neutral pH according to literature,^35^ may disfavor the formation of well-ordered micelle-type morphologies.

The aggregation propensity of **1** and **2** was also analyzed by DLS measurements in the less polar organic solvents methanol-*d*_4_ ^36^ and chloroform at 10 μM (Figure 3b). In methanol-*d*_4_, both C_60_-oligo-Lys **1** and C_60_-PEG **2** exhibit relatively larger particle size (mean diameters of ca. 50 and 80 nm, respectively, Figure 3b, middle) in comparison with water. Interestingly, when apolar chloroform is used as a solvent, **2** shows a significantly reduced particle size (with a mean value of ca. 3 nm), while **1** forms relatively larger aggregates (ca. 70 nm) (Figure 3b bottom). This indicates that micelle formation does not occur in a chloroform solution of **2**, and the molecules exist mainly as single units. This observation suggests that the solvent environment significantly influences the aggregation behavior of conjugates **1** and **2**, with solvent polarity playing a critical role in determining the particle size.

To obtain a deeper insight into the aggregation phenomena of C_60_-oligo-Lys **1** and C_60_-PEG **2** in aqueous solutions, a cryo-transmission electron microscopy (TEM) image of each solution was acquired (Figures 4 and S1). In this experiment, each solution of **1** or **2** in Milli-Q water (1 mM) was plunge-frozen and subjected to TEM imaging at a low temperature. As shown in Figure 4, both images show dark spots, presumably corresponding to C_60_ aggregates formed in the core of the micelle-type structures. When the images of **1** and **2** are compared, the aggregates appear relatively more prominent in the image of **2** (Figure 4, right), suggesting that the C_60_ core forms larger aggregates in the C_60_-PEG **2** micelles with respect to C_60_-poly-Lys **1**, the latter thus resulting in a well-dispersed aqueous solution. These characteristics of **1** are beneficial in PDT applications, as they are expected to reduce the self-quenching of C_60_ excited states, which often occurs when many PS molecules are confined at short distances as in the larger micelle morphologies found in **2**.

**Figure 4:**
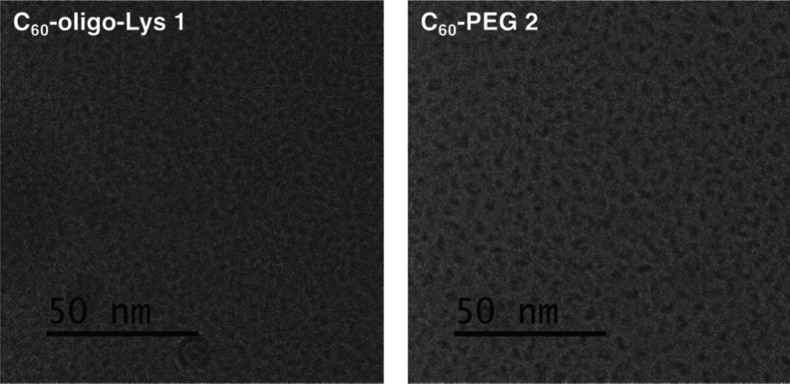
Cryo-TEM images of aqueous solutions of **1** (left) and **2** (right) (1 mM in Milli-Q water).

### Photoinduced ROS Generation by C_60_ Conjugates **1** and **2**

#### Pathways for ROS Generation under Visible Light Irradiation

Since the initial work by Foote and co-workers in 1991, it has been known that C_60_, excited by visible light, generates ^1^O_2_ via the type II energy transfer pathway with a unitary quantum yield in benzene (Scheme 1, type II energy transfer).^1^ Almost simultaneously, the same group reported the type I electron transfer reaction from an electron donor to the triplet excited state of C_60_ (^3^C_60_*), observing the formation of the C_60_ radical anion (C_60_^•–^) (Scheme 1, type I electron transfer).^37^ This reaction is facilitated by the relatively high first-electron-reduction potential estimated for ^3^C_60_* (*E*_1_ = +1.14 V vs SCE in PhCN)^37^ and proceeds quantitatively. These two types of photoreactions, observed in organic solvents, suggested that C_60_ possesses promising properties as a core for PDT–PS agents. A subsequent study by Nakamura in 1993 revealed photoinduced DNA cleaving activity by a water-soluble C_60_ carboxylic acid derivative, which generated ^1^O_2_.^14^ Following their work, we reported in 1998 the generation of O_2_^•–^ and ^•^OH by pristine C_60_ solubilized with PVP in aqueous media.^4^ Taken together with other reports by Foote and Rubin on direct reaction of C_60_^•–^ with DNA,^38^ and the higher quantum yield of ^1^O_2_ generation by C_60_ with respect to other PSs reported by Nagano,^3^ C_60_ has attracted further attention as a good PDT–PS candidate.

**Scheme 1:**
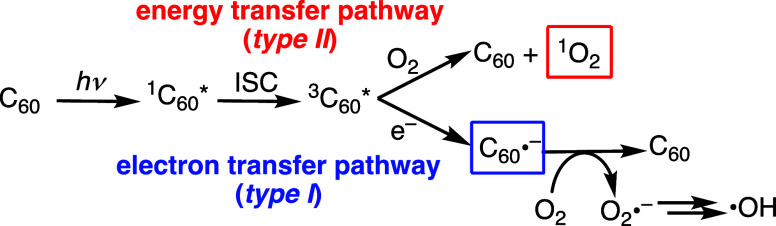
Photoinduced ROS Generation by C_60_ Derivatives via the Type II Energy Transfer or the Type I Electron Transfer Pathway

To improve the absorption properties at the desired phototherapeutic window, the use of C_70_, with a larger conjugated aromatic system, can be useful. In a recent study, we synthesized a water-soluble C_70_-PEG derivative and compared its ROS generation capacity with that of C_60_-PEG.^7^ Under visible light irradiation (527 nm), the generation of ^1^O_2_ by C_70_-PEG was significantly higher than that of C_60_-PEG. However, the generation of type I ROS (O_2_^•–^ and ^•^OH) was found to be more efficient in C_60_-PEG than in C_70_-PEG due to their redox potentials. It became our interest to investigate the effects of water-soluble moieties attached to the PS core on ROS generation by the entire molecule. In some of the previous studies, it has been shown that anionic derivatives of C_60_ perform better in generating ^1^O_2_,^39^ and cationic derivatives are more active in generating O_2_^•–^ and ^•^OH.^10,40^ In this study, in order to see the effect of oligo-Lys anchors appended to the C_60_ PS core, we explore the ROS generation of **1** under visible light irradiation (527 nm) with reference to the control C_60_-PEG **2**, considering both type II energy transfer and type I electron transfer pathways (Scheme 1).

#### ^1^O_2_ Generation

An ESR measurement for ^1^O_2_ generation was performed using 4-oxo-TEMP as a spin-trapping agent.^41^ Upon visible light irradiation (green LED with a maximum at 527 nm) in the presence of 4-oxo-TEMP, specific ESR signals corresponding to the 4-oxo-TEMPO radical are observed in the aqueous solutions of both **1** and **2** (Figures 5a,b, S2, and S3). The intensity of 4-oxo-TEMPO signals increases in an irradiation-time-dependent manner (Figures 5c, S2, and S3), clearly indicating that the generation of ^1^O_2_ is induced by the photoexcitation of the molecules. Interestingly, a significant difference between **1** and **2** in the amount of ^1^O_2_ generation is observed (ca. three times higher for **1**), despite both possessing the same PS core (C_60_) and the same substitution pattern at the [6,6]-junction with the fulleropyrrolidine skeleton (Figure 1).

**Figure 5:**
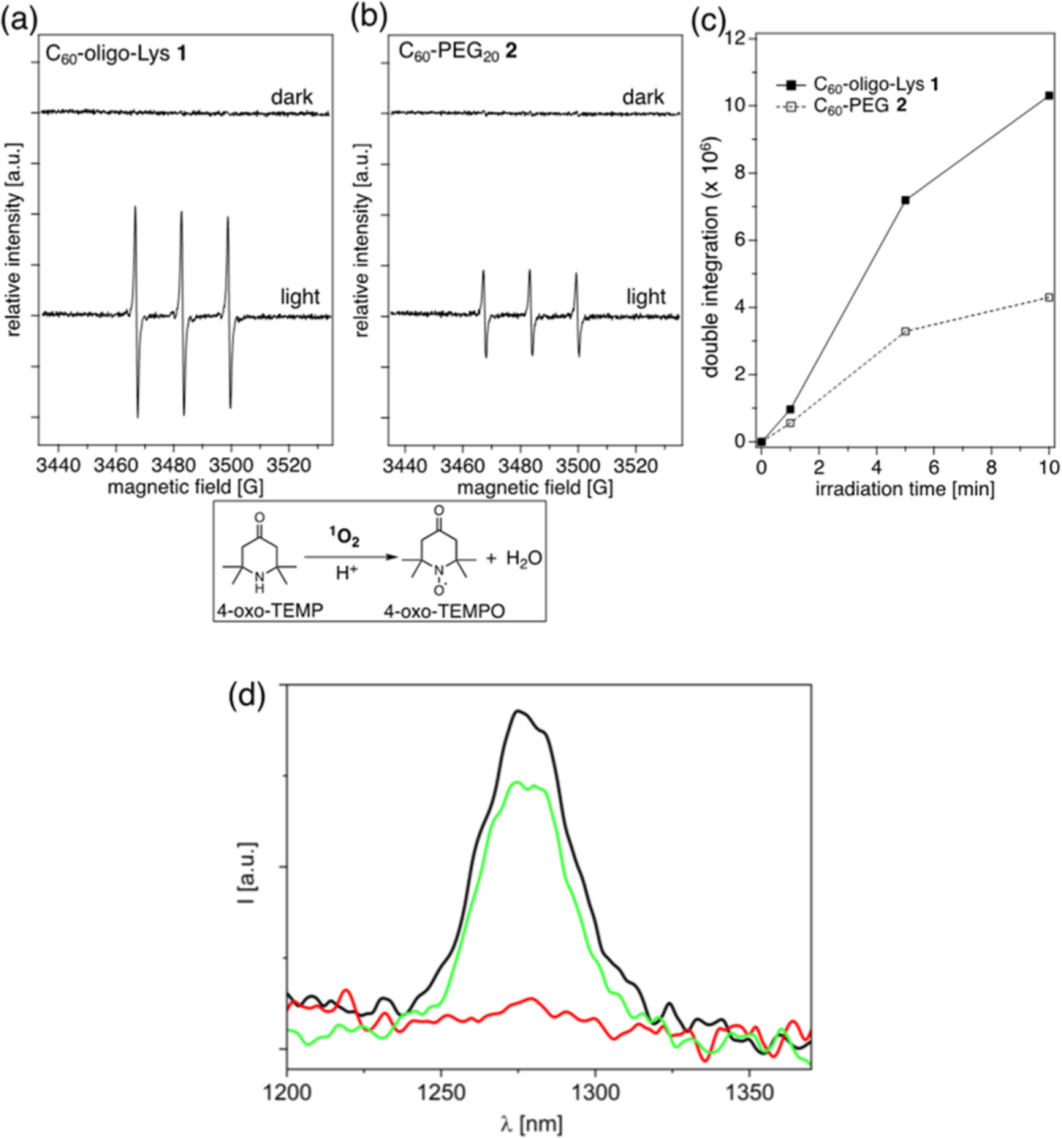
Light-induced ^1^O_2_ generation by C_60_-oligo-Lys **1** and C_60_-PEG **2**. (a,b) X-band ESR spectra of the 4-oxo-TEMP adduct with ^1^O_2_ generated in the aqueous solutions of **1** (a) and **2** (b) under irradiation with a green LED lamp (527 nm) for 10 min. C_60_ 40 μM; 4-oxo-TEMP 80 mM; and in 60 mM phosphate buffer (pH = 7.0). Measurement conditions: temperature 296 K; microwave frequency 10.03 GHz; microwave power 10 mW; receiver gain 5.0 × 10^4^; modulation amplitude 1.00 G; modulation frequency 100 kHz; sweep time 83.89 s; and scan times 10 times. (c) Irradiation time-dependent increase of double integration values corresponding to the 4-oxo-TEMPO signal generated in the aqueous solutions of C_60_-oligo-Lys **1** and C_60_-PEG **2**. C_60_ 40 μM; 4-oxo-TEMP 80 mM; and in 60 mM phosphate buffer (pH = 7.0). (d) ^1^O_2_ phosphorescence from optically matched D_2_O solutions of C_60_-oligo-Lys **1** and C_60_-PEG **2** (in oxygen-saturated conditions, black and red lines, respectively) and standard TPPS_4_ (in ambient conditions, green line); for all data, see Table S1. λ_exc_ = 325 nm and *A*_325_ = 0.69.

The quantum yields of ^1^O_2_ production in solutions of **1** and **2** were estimated by directly measuring the ^1^O_2_ phosphorescence at 1274 nm. As a standard PS, *meso*-tetra-(4-sulfonatophenyl) porphyrin (TPPS_4_, ϕ_Δ_ = 0.64) was used.^30^ Figure 5d shows the luminescence spectra of isoabsorbing oxygen-saturated D_2_O solutions of **1**, **2**, and TPPS_4_ upon excitation at 325 nm. It was observed that C_60_-oligo-Lys **1** exhibits a significant generation of ^1^O_2_ (black line, ϕ_Δ_ = 0.71), while, in the case of C_60_-PEG **2**, the intensity is too weak to determine a yield (red line, at the limit of experimental resolution, ϕ_Δ_ ≤ 0.1). These results are in line with the data obtained with the ESR spin-trapping method described above, although in this case the difference in ^1^O_2_ generation efficiency among the two compounds is found to be much higher. This discrepancy can be explained by the use of a spin-trapping agent in the ESR experiment that could partially prevent aggregation.

It is known that, in aggregated forms, the excited states of C_60_ can undergo annihilation, resulting in a decrease of ^1^O_2_ production.^42,43^ In addition, molecular oxygen may have difficulties to access the sheltered ^3^C_60_* core in **2** due to the shielding effect of the attached PEG anchors in the outer shell of the micelle.^20^ Furthermore, ^1^O_2_ generation by **1** and **2** is found to be similar in methanol-*d*_6_ (Figure S4), where the two conjugates present a similar aggregation behavior, as observed by DLS (Figure 3b, middle). ^1^O_2_ generation by **2** is much higher than that for **1** in chloroform solutions (Figure S5), where the aggregation tendency is higher for **1** as indicated by DLS (Figure 3b, bottom). On these bases, and taking together DLS and cryoTEM data (Figures 3 and 4), we can rationalize the decreased ^1^O_2_ generation by **2** with respect to **1** in aqueous solutions. This was further confirmed by transient absorption analysis (vide infra).

#### O_2_^•–^ and ^•^OH Generation

O_2_^•–^ is an essential intermediate in the photoinduced oxidative damage of biomolecules by the type I electron transfer pathway (Scheme 1). Via subsequent Fenton reaction, O_2_^•–^ is converted to a stronger ROS, ^•^OH,^44^ which can directly react with biomolecules (e.g., DNA, lipids, and proteins) further resulting in cell damage. To evaluate **1** and **2** as potential PDT–PSs, visible light-induced O_2_^•–^ generation was measured by the ESR spin-trapping method using DEPMPO as a spin-trapping reagent.^45^ DEPMPO is known to react with O_2_^•–^ to form DEPMPO–OOH as a relatively stable radical species, revealing specific signals observed by the ESR. To trigger the electron transfer pathway, NADH was added as an external electron donor to the system. In the type I pathway, ^3^C_60_*, with a relatively higher redox potential, is expected to easily receive an electron from the electron donor molecules to form O_2_^•–^. On the other hand, in the type II pathway, ^1^O_2_, once generated by the energy transfer process, can also receive an electron to form O_2_^•–^. To strictly differentiate these two pathways (via the type I and via the type II), the measurements were carried out also in the presence of l-histidine, a ^1^O_2_ quencher (Scheme 2). By adding l-histidine, O_2_^•–^ solely arising from the type I mechanism is expected to be detected.

**Scheme 2:**
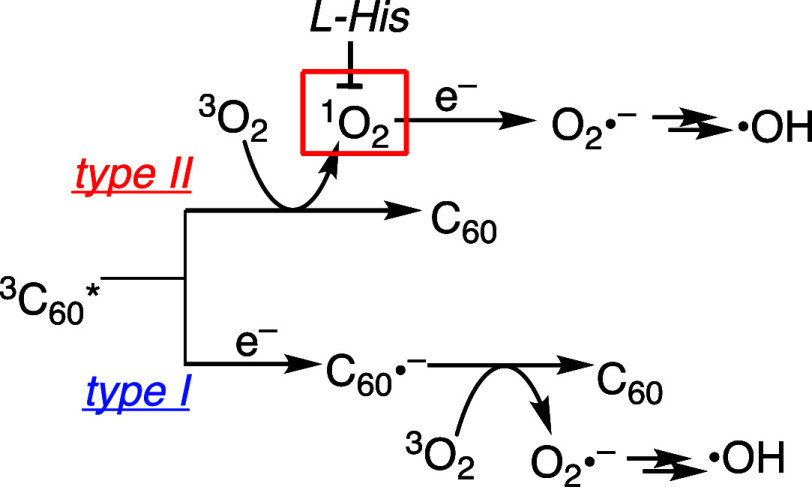
Two Potential Pathways in the Generation of Superoxide (O_2_^•–^) and Hydroxyl Radical (^•^OH) from Photoexcited C_60_ via the Type II Energy Transfer Pathway and Type I Electron Transfer Pathway

As reported in Figure 6a, in the presence of NADH as an e^–^ donor and under visible light irradiation, generation of O_2_^•–^ by C_60_-oligo-Lys **1** is clearly observed as the ESR signals corresponding to DEPMPO–OOH (Figure 6a, top black spectrum). In contrast, the generation of O_2_^•–^ by C_60_-PEG **2** is observed but with a much lower signal intensity under the same conditions (Figure 6b, top black spectrum). Higher O_2_^•–^ generation by **1** than **2** may be, in part, due to the effect of aggregation on **2**, as also observed in ^1^O_2_ generation. Notably, the generation of the O_2_^•–^ by **1** is observed even without the addition of an e^–^ donor (Figure 5a, bottom blue spectrum, and Figure S6c). This can be explained by the presence of partially unprotonated amine moieties in the oligo-Lys anchors of **2** at pH 7, which may act as electron donors, triggering the intramolecular electron transfer reaction to form C_60_^•–^. The generation of O_2_^•–^ is clearly shown in an irradiation-time-dependent manner for both **1** and **2** (Figures 6c,d and S6a,b).

**Figure 6:**
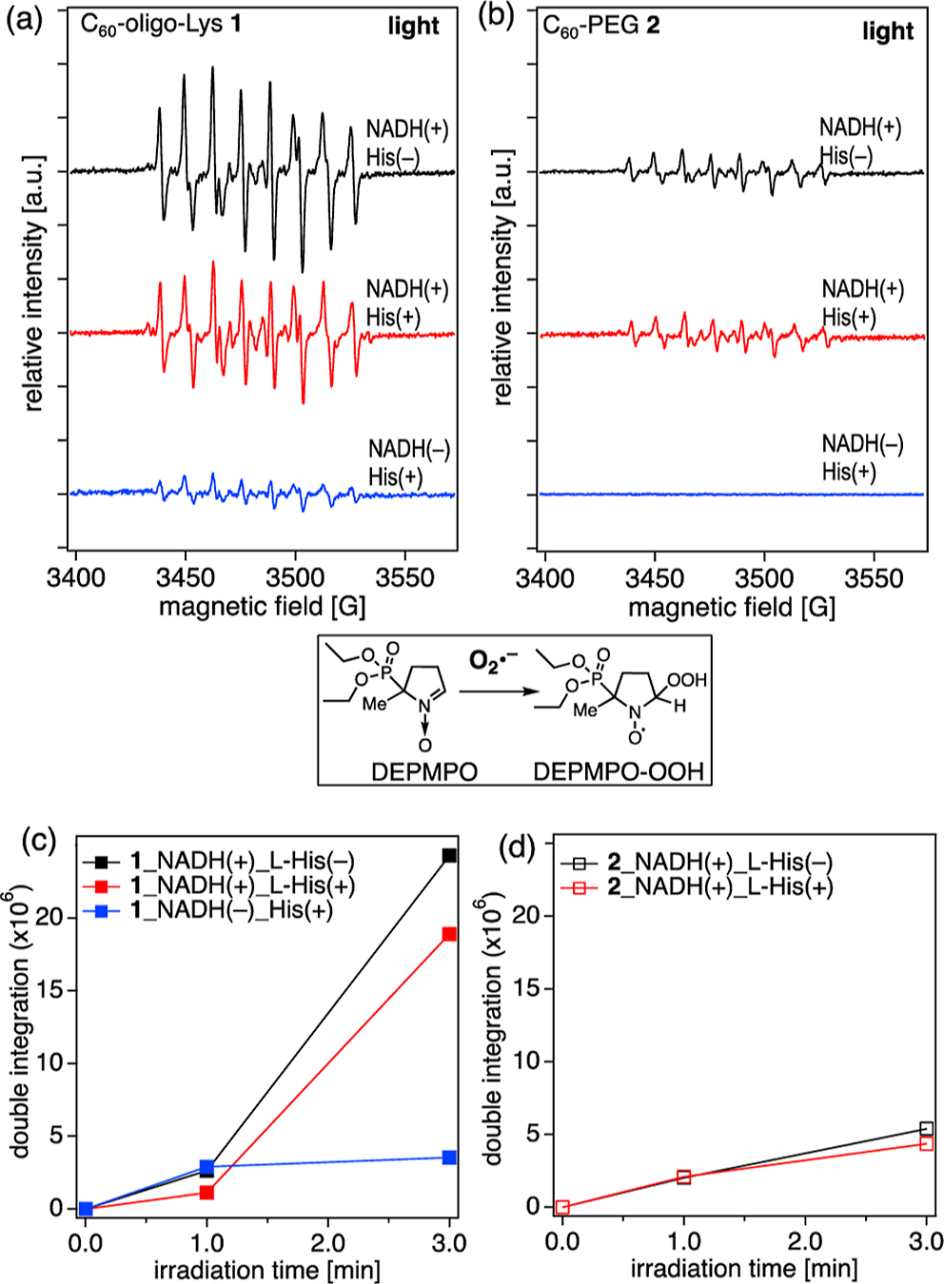
(a,b) X-band ESR spectra of the DEPMPO adduct with O_2_^•–^ generated in aqueous solutions of C_60_-oligo-Lys **1** (a) and C_60_-PEG **2** (b) under irradiation with a green LED (527 nm) for 3 min. C_60_: 40 μM, DEPMPO: 113 mM, NADH: 10 mM, DETAPAC: 1 mM, and l-histidine: 10 mM in 60 mM phosphate buffer (pH = 7.0) with 20% DMSO (v/v). Measurement conditions: temperature 296 K; microwave frequency 10.03 GHz; microwave power 10 mW; receiver gain 5.0 × 10^4^; modulation amplitude 1.00 G; modulation frequency 100 kHz; sweep time 83.89 s; and scan times 10 times. (c,d) Irradiation time-dependent generation of O_2_^•–^ by C_60_-oligo-Lys **1** (c) and C_60_-PEG **2** (d), estimated by double integration of the peaks corresponding to DEPMPO–OOH.

In the presence of l-histidine, a ^1^O_2_ quencher, a slight decrease in O_2_^•–^ generation (ca. 23%) is observed for C_60_-oligo-Lys **1** (Figure 6a, middle red spectrum). This difference should correspond to the amount of O_2_^•–^ generated via ^1^O_2_ once generated by the Type II mechanism (Scheme 2). Conversely, a faint decrease in O_2_^•–^ generation is observed in the case of C_60_-PEG **2** (Figure 6b, middle red spectrum), in line with the limited amount of ^1^O_2_ produced from **2**, as described in the section above.

The generation of ^•^OH, a strong ROS, was confirmed by ESR using DMPO as a spin-trapping reagent. In the presence of an electron donor (NADH), an efficient ^•^OH generation by **1** is detected, as indicated by signals corresponding to DMPO–OH (Figure 7a­(i)). Similar to the O_2_^•–^ generation, ^•^OH production by **1** is observed even in the absence of NADH (Figure 7a­(ii)). The generation of ^•^OH in C_60_-PEG **2** is observed only in the presence of NADH, although the signal intensity is much lower in comparison to the case of **1** (Figure 7a­(iii)), as is clearly shown also in the irradiation-time-dependent results reported in Figure 7b.

**Figure 7:**
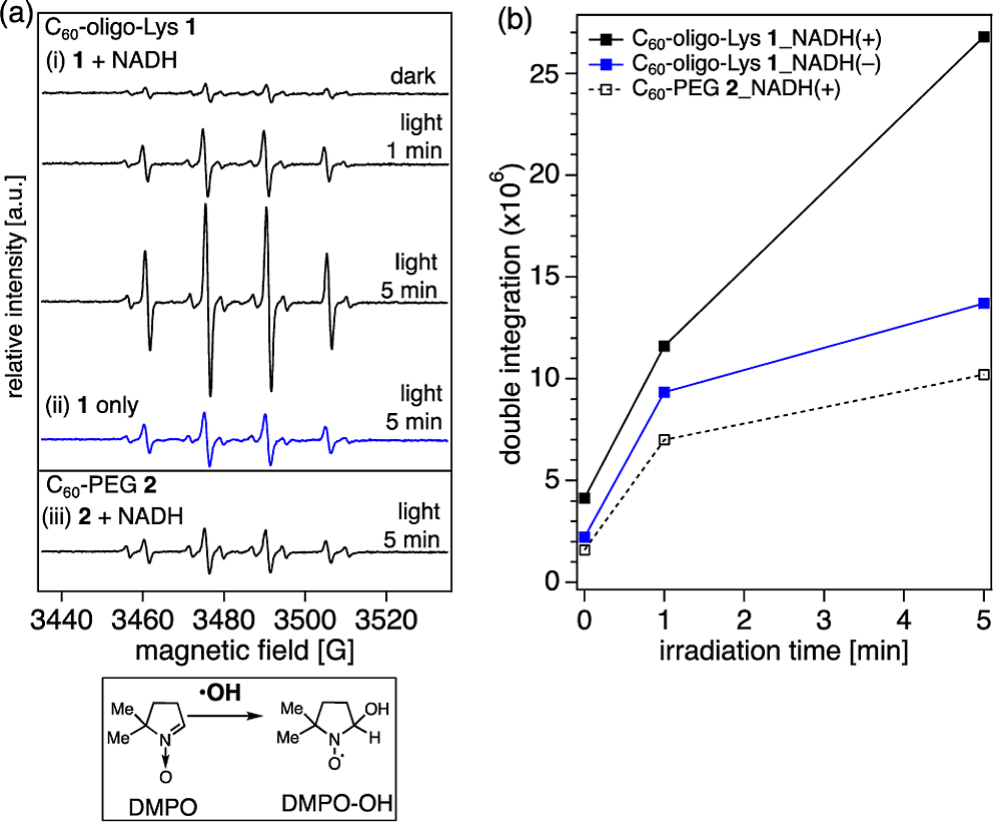
(a) X-band ESR spectra of the DMPO adduct with ^•^OH generated in C_60_-oligo-Lys **1** (i,ii) and C_60_-PEG **2** (iii) in aqueous solutions under irradiation with a green LED lamp (527 nm) for 5 min. C_60_ 40 μM, DMPO 145 mM, NADH 10 mM, and Fe­(II)-DETAPAC 40 μM in 60 mM phosphate buffer (pH = 7.0). Irradiation time: 0, 1, and, 5 min. Measurement conditions: temperature 296 K; microwave frequency 10.03 GHz; microwave power 10 mW; receiver gain 5.0 × 10^4^; modulation amplitude 1.00 G; modulation frequency 100 kHz; sweep time 83.89 s; and scan times 10 times. (b) Irradiation time-dependent generation of ^•^OH estimated by double integration of the ESR signals corresponding to DMPO–OH.

Overall, the data provides evidence of a clear difference between C_60_-oligo-Lys **1** and C_60_-PEG **2** in ROS generation, with **1** being much more efficient in producing ^1^O_2_, O_2_^•–^, and ^•^OH, indicating that the C_60_-oligo-Lys conjugate **1**, developed in this study, is a more suitable PS for PDT.

### Transient Absorption Spectroscopy

To better understand the excited state dynamics of conjugates **1** and **2** following photoexcitation in water, transient absorption measurements on ultrafast and fast time scales (300 fs to 3 ns) have been performed (Figure 8a). As a suitable model for the interpretation of the transient signals of the conjugates, fulleropyrrolidine monoadduct **3** (Figure 1) was investigated in comparison to C_60_-PEG **2** in chloroform (Figure 8b) since **2** and **3** are thoroughly soluble in this solvent or do not present significant aggregation.

**Figure 8:**
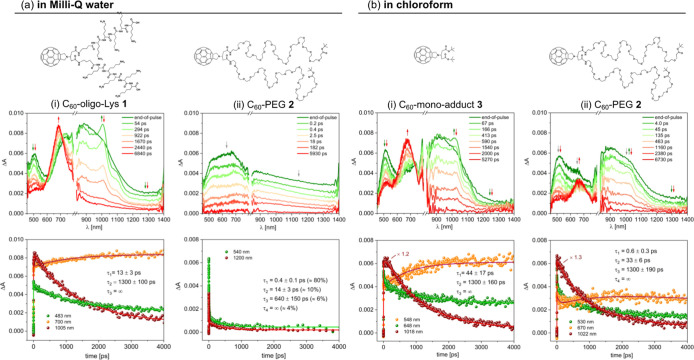
Transient absorption spectra (upper raw) and Δ*A* evolution at significant wavelengths (lower raw) of conjugates **1** and **2** in Milli-Q water (pH = 7.0) (a) and of model **3** and conjugate **2** in chloroform (b). λ_exc_ = 320 nm, *A*_320_ = 0.2, 0.2 cm optical path, and *E* = 4 μJ/pulse.

In water, conjugate **1** shows an initial Δ*A* spectrum characterized by a peak at 500 nm and a large band centered at ca. 900 nm (Figure 8a­(i), top). This signal evolves with a biphasic behavior: (1) first, a fast process, on the order of 13 ps, leads to the decrease of the band at 500 nm and to the formation of a peak at 1005 nm; (2) the second process is characterized by the decay of all bands in ca. 1.3 ns and, concomitantly, by the rise of a band at 690 nm that remains stable on longer time scales (the kinetics are reported in Figure 8a­(i), bottom). Interestingly, model **3** in chloroform shows exactly the same behavior (Figure 8b­(i)), with slight differences in the peak maxima (1018 and 680 nm for the two formed peaks) and in the lifetime of the first process (44 ps vs 13 ps), reasonably due to the different solvent. The band at ca. 700 nm, with an “infinite” lifetime on the time scale of the experiment, can be unequivocally ascribed to the triplet excited state of the fulleropyrrolidine core.^46,47^ Its formation occurs within 1.3 ns, matching the measured fluorescence lifetime (Table 1), confirming that the longer process is the intersystem crossing from the fullerene singlet. The initial fast process of singlet formation can be attributed to a solvent-induced vibrational relaxation, and the band at 1005 nm, forming in this process, is attributed to the fulleropyrrolidine singlet. Noteworthily, the position of this peak closely resembles that of the fulleropyrrolidine anion, reported at around 1000–1010 nm from spectroelectrochemical data or transient absorption analysis of multichromophoric systems undergoing photoinduced electron transfer reactions.^47–53^ However, here the peak can be undoubtedly ascribed to the singlet excited state of the fulleropyrrolidine unit, likely exhibiting a charge transfer nature due to the presence of the pyrrolidine donor group. To the best of our knowledge, this feature is reported here for the first time since ultrafast transient absorption studies on fulleropyrrolidines not incorporated in multicomponent arrays are rare.^54^

For conjugate **2** in water, the scenario is completely different: a broad and featureless spectrum covers the entire vis–NIR spectral range and decays very quickly (Figure 8a­(ii)). The kinetics, indeed, is multiexponential, with a subpicosecond component (0.4 ps) dominating the decay. It can be noticed that the multiexponential fitting matches that observed for the emission decay on the ns scale (Table 1), thus confirming that the quenching of the singlet excited state of the fullerene core arises from annihilation processes due to aggregation. When **2** was examined in chloroform, a solvent where aggregation is strongly reduced (see the DLS data in Figure 3b, bottom and related discussion), the processes of singlet formation and population of the triplet are again observable, but they are preceded by a fast decay (0.6 ps) of the entire initial spectrum (Figure 8b­(ii)). This behavior can be ascribed to the overlay of signals arising from monodispersed and aggregated molecules, the latter presumably constituting a small fraction of the whole population.

Overall, these results provide evidence that, in water, the formation of the fullerene triplet excited state is detectable only for the non-aggregated compound **1**. As a matter of fact, this state is the fundamental species engaged in the energy and electron transfer bimolecular processes occurring on longer time scales, which accounts for the higher efficiency of **1** with respect to **2** in ROS generation.

### Photocytotoxicity

#### Photocytotoxicity of **1** and **2**

The results described above clearly demonstrate that C_60_-oligo-Lys **1**, with less aggregation propensity in an aqueous environment, generates ROS (^1^O_2_, O_2_^•–^, and ^•^OH) more efficiently than C_60_-PEG **2**, suggesting that **1** will be a more suitable PS core for PDT application. To further investigate the properties of **1** and **2** as PDT–PSs, in vitro photocytotoxicity tests were performed on HeLa cells by a standard 3-(4,5-dimethylthiazol-2-yl)-2,5-diphenyltetrazolium bromide (MTT) method.^55^ Before the assay, it was confirmed that **1** and **2** have no dark cytotoxicity (IC_50_ > 25 μM, Figure 9a, gray lines).

**Figure 9:**
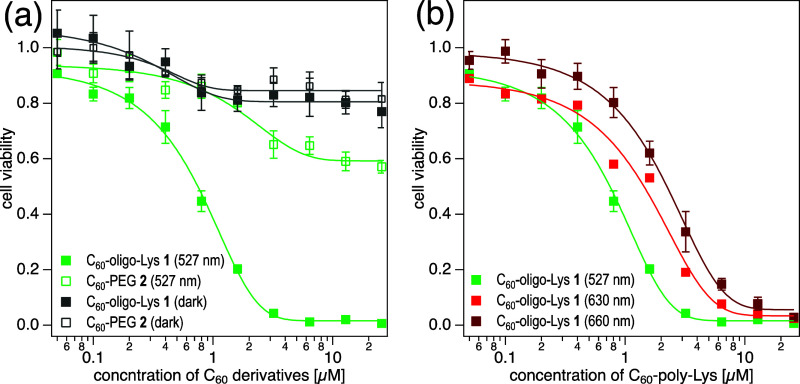
(a) Photocytotoxicity of C_60_-oligo-Lys **1** and C_60_-PEG_20_ **2** on HeLa cells upon green LED irradiation (527 nm, 25 mW per well). (b) Effect of wavelength on the photocytotoxicity of C_60_-oligo-Lys **1**. Green LED (527 nm, 25 mW per well), red LED (630 nm, 25 mW per well), or dark-red LED (660 nm, 35 mW per well) was used. Irradiation time: 15 min. Cell viability assay: MTT assay.

The cells were incubated in the presence of C_60_ conjugate **1** or **2** for 24 h and subsequently washed with PBS(−) before being exposed to the LED light (with a maximum at 527 (green), 630 (red), or 660 (dark red) nm) for 15 min. Cell viability was evaluated by the MTT assay. The results shows that the viability of the cells incubated with C_60_-oligo-Lys **1** is drastically decreased by the irradiation with green light (527 nm) in a concentration-dependent manner (Figure 9a, green closed squares, IC_50_ ca. 0.8 μM), while C_60_-PEG **2** exhibits only a limited photocytotoxicity (Figure 9a, green open squares, IC_50_ > 25 μM). The photocytotoxicity of **1** is also observed upon irradiation with light with longer wavelengths (630 and 660 nm) with a slight increase of IC_50_ (Figure 9b). Taking into account that these longer wavelengths are more suitable for use in PDT due to their better tissue penetration, considerable photocytotoxicity of **1** shown here suggests a high potential of **1** as a PDT–PS candidate.^56–61^

#### Effects of ROS Quenchers on Photocytotoxicity by **1**

The aforementioned efficient photocytotoxicity of **1** was expected to be related to enhanced ROS generation by **1** as described in the previous sections. To obtain more direct evidence of the involvement of ROS in the photocytotoxicity, the same tests were performed for **1** in the presence of several ROS scavengers.

To confirm the absence of significant cytotoxicity of the quenchers themselves, the cells were treated only with quenchers without **1** in the control experiment (Figure 10, left, **1**: 0 μM). The cells, treated with **1** (at 3.2 or 6.4 μM, which have shown sufficient photocytotoxicity as shown in Figure 9a), were irradiated by visible light (527 nm) in the presence of NaN_3_ (a ^1^O_2_ quencher), superoxide dismutase (SOD, a O_2_^•–^ quencher), or mannitol (a ^•^OH quencher). In the presence of quenchers, the viability of cells significantly recovered in both groups treated with **1** at 3.2 (Figure 10, middle) and 6.4 μM (Figure 10, right). The results indicate that all ROS (^1^O_2_, O_2_^•–^, and ^•^OH) are involved in the photocytotoxicity of **1** to some extent, in line with the ESR results described above, indicating the generation of all types of ROSs by **1** under visible light irradiation in the in vitro biological systems to significantly damage the cells.

**Figure 10:**
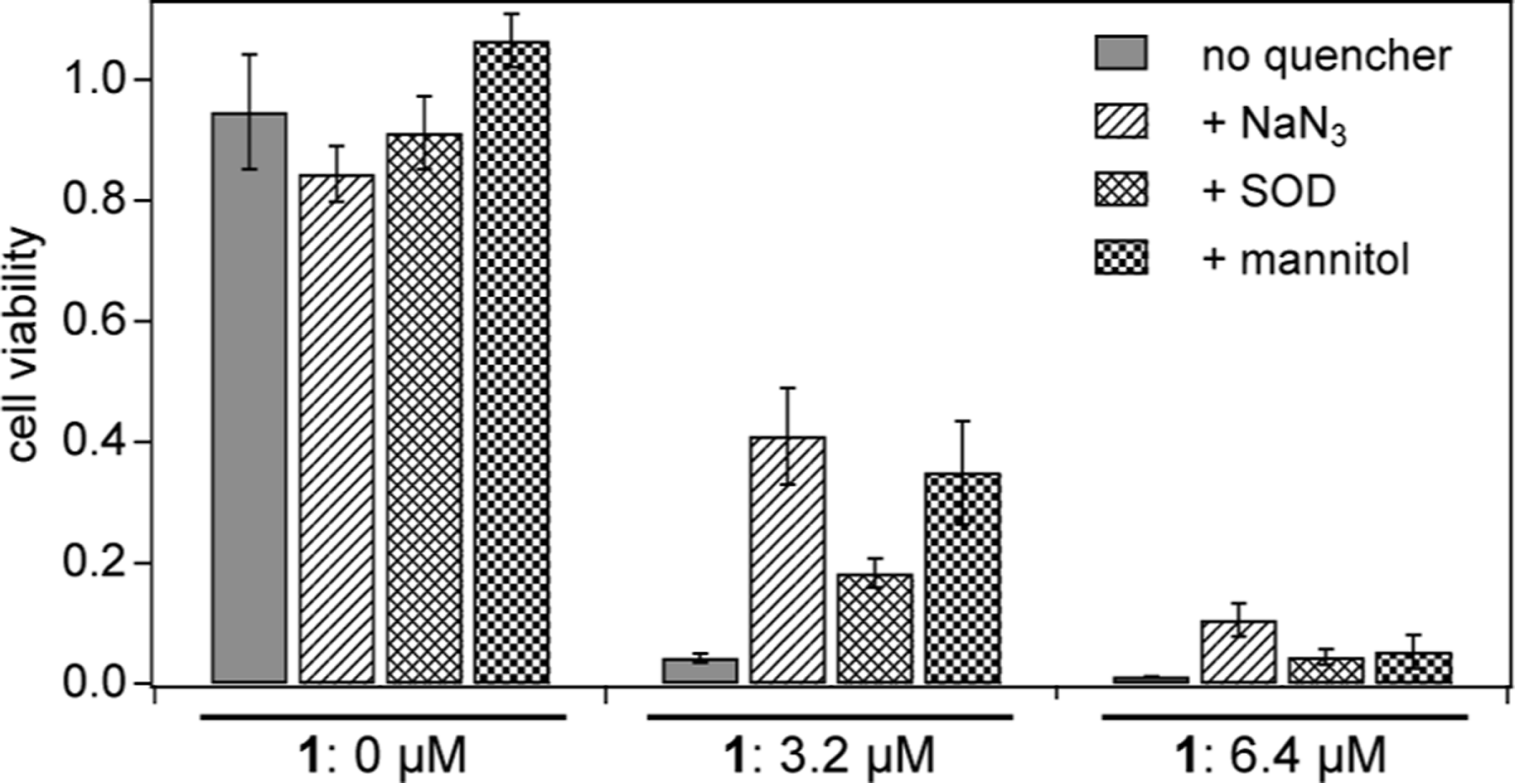
Effect of ROS quenchers on the photocytotoxicity of C_60_-oligo-Lys **1** (0, 3.2, and 6.4 μM). Concentrations of the quenchers: NaN_3_ 10 mM, SOD 0.5 unit mL^–1^,^7^ and mannitol 10 mM. Irradiation conditions: green light (527 nm, 25 mW per well) for 15 min. Cell viability was measured by the MTT assay and using HeLa cells.

#### Comparison to Previously Reported Data

Previous studies of ROS generation by water-soluble C_60_-based PSs were performed using C_60_ derivatives with anionic and cationic anchors. For instance, cationic derivatives such as hexa­(sulfobutyl)­fullerene generated ^1^O_2_ efficiently, while no type I ROS were observed.^62,63^ A similar situation was observed in our previous study on the C_60_-carboxylate derivative.^6^ On the other hand, the cationic C_60_ tris-functionalized *N*,*N*-dimethylfulleropyrrolidine derivatives,^61^ and the decacationic C_60_ monoadduct,^40,64^ revealed photocytotoxicity involving the generation of both ^1^O_2_ and O_2_^•–^ on several cancer cell lines. In these reported cases, the electron transfer reaction leading to the production of O_2_^•–^ is an intermolecular process involving external electron donors (NADH or iodide counter anion). Noteworthily, in the present case of the C_60_-oligo-Lys derivative **1**, the electron transfer occurs also intramolecularly, from the amine residues of the oligo-Lys chains to the fullerene core.

Among numerous synthetic dyes explored as PSs, few examples demonstrate the ability to generate ROS through type I electron transfer reactions. These ROS species, capable of biomolecule degradation at low oxygen concentrations, can be efficient therapeutic agents, especially in the hypoxic tumor microenvironment.^65–67^ Our recent results indicate that C_60_ can undergo type I electron transfer faster than the similar fullerene C_70_, resulting in more efficient DNA photocleavage, despite the higher absorption of C_70_ in the visible region.^7^ The efficient type I ROS generation by **1** in comparison to C_60_-PEG **2** shown in this study indicates its excellent ability to serve as a PDT–PS due to (i) reduced aggregation that offers enhanced ROS production and (ii) the possibility of intramolecular electron transfer processes.

## Conclusions

Two types of water-soluble C_60_ derivatives, C_60_-oligo-Lys conjugate **1** and C_60_-PEG conjugate **2**, synthesized from a Prato adduct of C_60_ **3**, were evaluated as potential PDT–PSs. Conjugates **1** and **2** were characterized on the basis of their aggregation phenomena and associated photophysical properties in aqueous solutions. The absorption and emission features, DLS, and cryoTEM results indicate significant differences in the size of the aggregates among **1** and **2** bearing different anchors. The ^1^O_2_ generation by **1** and **2** was evaluated by both the ESR spin-trapping method and ^1^O_2_ phosphorescence detection, indicating that the production of ^1^O_2_ is decreased in **2** due to a lager extent of aggregation. Furthermore, **1** exhibits a more efficient generation of O_2_^•–^ and ^•^OH, which work as stronger ROS in PDT, by electron transfer reactions via C_60_^•–^. Transient absorption measurements do not provide evidence of the formation of the fulleropyrrolidine anion of **1** in water on a ps time scale but show the population of the triplet state, which is decreased in **2** due to annihilation processes occurring in the aggregated species. Finally, **1** shows enhanced photocytotoxicity on Hela cells due to its superior ROS generation capacity. The results demonstrate that the highly water-soluble C_60_-oligo-Lys derivative **1** reported here is a good candidate with improved properties for a C_60_-based PDT.

## Supplementary Material



## Notes

Details of absorption and emission measurements; DLS measurements; cryoTEM imaging; ^1^O_2_ quantum yield measurement by phosphorescence; ESR measurements for generations of ^1^O_2_, O_2_^•–^, and ^•^OH; transition absorption spectroscopy; and photocytotoxicity assay (PDF)
