# Supplementary material for: A Highly Water-Soluble C60-Oligo-Lysine Conjugate as a Type I and Type II Photosensitizer with Enhanced ROS Generation and Photocytotoxicity
Source: ACS Phys Chem Au. 2025 May 27;5(4):398–409. doi: 10.1021/acsphyschemau.5c00023 (PMC12291136; doi:10.1021/acsphyschemau.5c00023)
Supplement: Supplementary file 1 [file pg5c00023_si_001.pdf]

A Highly Water-Soluble C_{60}-Oligo-Lysine Conjugate as a Type I and Type II Photosensitizer with Enhanced ROS Generation and Photocytotoxicity

Yue Ma,[a] Lorenzo Persi,[a] Kateryna A. Tolmachova,[a] Maxim Yulikov,[a] Miroslav Peterek,[b] Stephan Handschin,[b] Nicola Armaroli,[c] Barbara Ventura*[c] and Yoko Yamakoshi*[a]

[a] *Department of Chemistry and Applied Biosciences, ETH Zürich, Vladimir-Prelog-Weg 3, CH-8093 Zürich, Switzerland. E-mail: yamakoshi@org.chem.ethz.ch*

[b] *ScopeM, ETH Zürich, Otto-Stern-Weg 3, CH-8093 Zürich, Switzerland*

[c] *Istituto ISOF-CNR, Via P. Gobetti 101, 40129 Bologna, Italy. E-mail: barbara.ventura@isof.cnr.it*

- Supporting Information-

1. DLS

DLS measurements were performed on a Malvern Nano-ZetaSizer (Malvern Instruments Ltd., Worcestershire, UK) equipped with a 5 mW HeNe laser (wavelength: 632.8 nm) and a digital logarithmic correlator. C_{60}-oligo-Lys **1** and C_{60}-PEG **2** solutions were prepared in milli-Q water (pH=7.0) with concentrations of 10 µM, 40 µM, and 1 mM. For the measurements in chloroform and methanol, 10 µM solutions of C_{60}-oligo-Lys **1** and C_{60}-PEG **2** were prepared. All the measurements were performed at 25 ºC.

2. CryoTEM

An aliquot of 1 mM C_{60}-poly-Lys **1** or C_{60}-PEG **2** (3.4 µl each) was added onto lacey carbon-coated copper grids (Electron Microscopy Sciences, Hatfield, PA, USA), or on holey carbon grids coated with thin carbon (R2/2 + 2nmC, Quantifoil Micro Tools GmbH, Jena, Germany), which were previously negatively glow-discharged (Emitech K100X, GB). Excess of sample was blotted away for two or two and a half seconds (for quantifoil grids) using a Vitrobot Mark IV (Thermo Fisher Scientific, USA) with the environmental chamber set to 22 °C and 100% humidity and the grids were plunge frozen in a mixture of liquid ethane/propane (continuously cooled by liquid nitrogen). Vitrified grids were stored in liquid nitrogen until further processing.

The cryo-EM grids were loaded into a Titan Krios microscope operating at 300 kV (Thermo Fisher Scientific, USA), equipped with a Gatan Quantum-LS Energy Filter and a Gatan K2 Summit direct electron

detector (Gatan Inc., Pleasanton, CA, USA). The samples were imaged in EFTEM mode using the Thermo Fisher Scientific EPU software (215000x magnification, approx. 60 e-/A2 total electron dose, K2 in linear mode), with a defocus range of –2 to –4 μm. Resulting micrographs were saved in ".tiff" format using the DigitalMicrograph Software from Gatan Microscopy Suite (GMS).

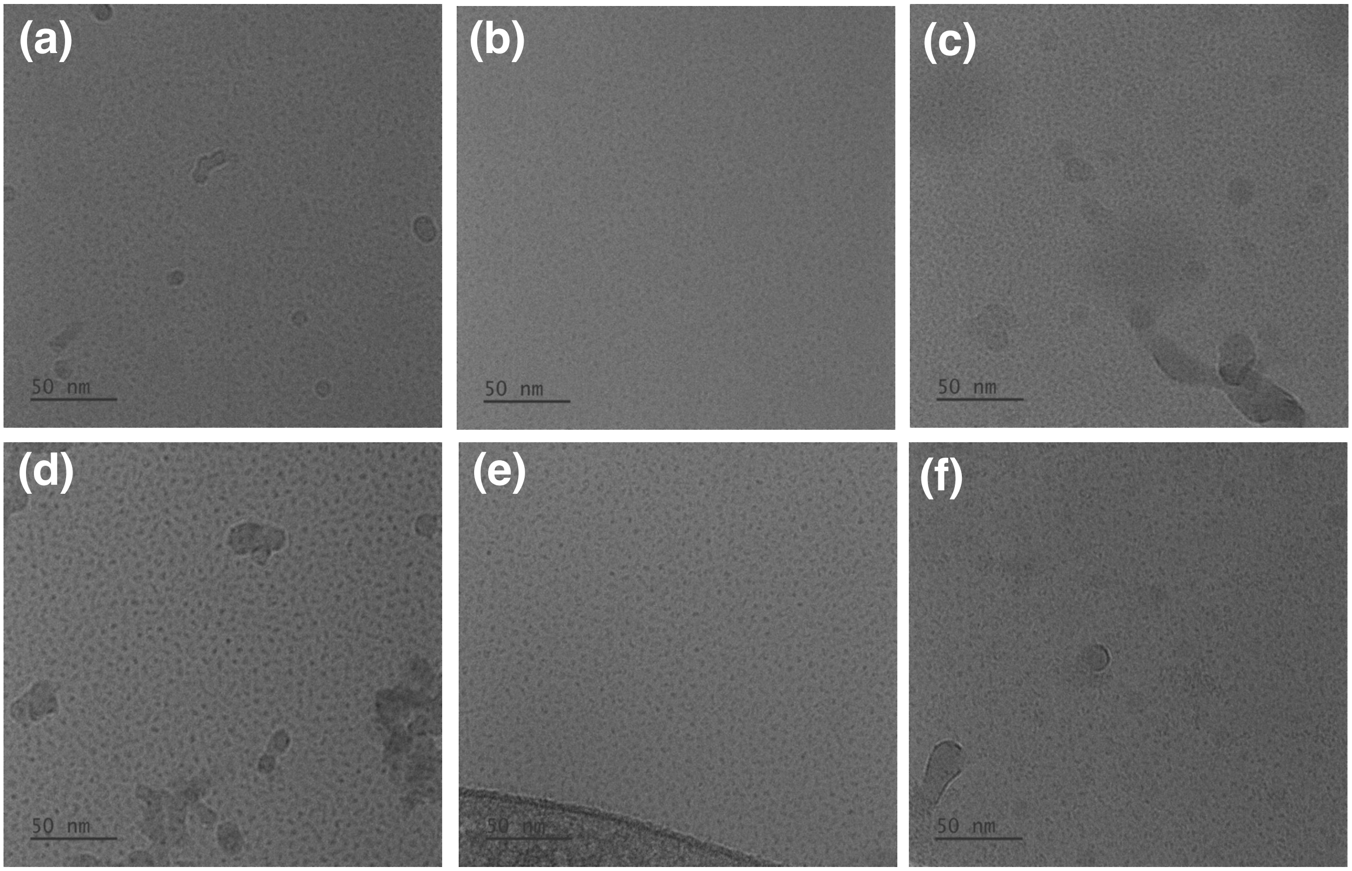

Figure S1. CryoTEM micrographs of C_{60}-poly-Lys **1** (a-c) and C_{60}-PEG **2** (d-f) in aqueous solution (1 mM) on lasy grids (a, c, d, e) and on quantifoil holey grids with a thin carbon layer (c and f).

3. Absorption and emission spectroscopy, photophysics

Solutions of **1** and **2** were prepared in tridistilled Milli-Q water (Sartorius Arium® Ultrapure water system, Göttingen, Germany), with pH adjusted to 7.0. D_2O was purchased from VWR Chemicals (VWR International, LLC, Leuven, Belgium) and C_{60} and $TPPS_4$ from Sigma-Aldrich (St. Louis, MO, USA), and used as received.

Absorption spectra were recorded with a PerkinElmer Lambda 950 UV-Vis-NIR spectrophotometer (PerkinElmer, Inc., Shelton, CT, USA) in 1 cm quartz cuvettes both with and without the use of a 100 mm integrating sphere. Emission spectra were collected in right-angle setup with both a FLS920

spectrofluorimeter (Edinburgh Instruments Ltd, Livingston, UK), equipped with a Peltier-cooled R928 PMT (280–850 nm) (Hamamatsu Photonics, Shizuoka, Japan) and an Edinburgh FLS920 fluorimeter equipped with a Hamamatsu R5509-72 InP/InGaAs photomultiplier tube supercooled at 193 K in a liquid nitrogen cooled housing and a TM300 emission monochromator with a NIR grating blazed at 1000 nm (300-1700 nm). The spectra have been corrected for the wavelength dependent phototube response. Fluorescence quantum yields have been determined with reference to C_{60} in aerated toluene ($\phi_{fl} = 2.2 \times 10^{-4}$)[1] upon excitation at 340 nm. Fluorescence lifetimes have been measured with an IBH Time Correlated Single Photon Counting apparatus with nanoLED excitation at 331 nm. The analysis of the luminescence decay profiles against time was accomplished with the DAS6 Decay Analysis Software provided by the manufacturer. The estimated error on molar absorption coefficients, luminescence lifetimes and quantum yields is 10%.

4. Singlet oxygen quantum yield determination by luminescence method

Singlet oxygen production quantum yields of **1** and **2** in D_2O solution has been measured with reference to 5,10,15,20-tetrakis(4-sulphonatophenyl)-porphyrin ($TPPS_4$) ($\phi_\Delta = 0.64$),[2] by comparing the intensity of singlet oxygen phosphorescence spectra, measured with the NIR fluorimeter described above, from optically matched solutions. D_2O has been used as a solvent to take advantage of the longer lifetime of $^1O_2$ in D_2O with respect to water.[3] Excitation at 325 nm has been performed with a HeCd laser (Kimmon Koha Co., Ltd., Tokyo, Japan). To obtain oxygen-saturation conditions, the D_2O solutions of the compounds were bubbled with pure oxygen for 5 minutes in custom gastight fluorescence cells. The data obtained in both ambient condition and oxygen saturation are collected in Table S1.

Table S1. Singlet oxygen production quantum yields in D_2O at different oxygenation conditions.

compounds	ϕ_Δ
C_{60}-oligo-Lys **1** (O_2-saturated solution)	0.71
C_{60}-oligo-Lys **1** (ambient conditions)	0.54
C_{60}-PEG **2** (O_2-saturated solution)	< 0.1
C_{60}-PEG **2** (ambient conditions)	< 0.1

5. ESR spin-trapping methods

ESR measurements were carried out on a Bruker spectrometer (Bruker BioSpin, GmbH, Rheinstetten, Germany), equipped with a microwave bridge X-band ER. Diethylenetriaminepentaacetic acid (DETAPAC), NADH, dimethyl sulfoxide (DMSO), $FeSO_4$, and L-histidine were purchased from Sigma-Aldrich Co. (St. Louis, MO, USA). Five-(diethoxyphosphoryl)-5-methyl-1-pyrroline-*N*-oxide (DEPMPO) was purchased from Enzo Life Sciences Inc. (Farmingdale, NY, USA). The 2,2,6,6-tetramethyl-4-piperidone (4-oxo-TEMP), and 5,5-dimethyl-1-pyrroline *N*-oxide (DMPO) were purchased from Tokyo Chemical Industry Co., Ltd. (Tokyo, Japan). All chemicals were used as purchased except for 4-oxo-TEMP, which was purified by sublimation before use. Photoirradiation was performed by Lumitronix PowerBar V3 true green LED (527 nm, 93 lm•W^{-1}, Osram Oslon SSL 150, Lumitronix LED-Technik GmbH, Hechingen, Germany) with a total of 160 lamps that were assembled in a cylindrical manner. The individual sample was loaded and sealed in a glass capillary (50 µL micropipette, Blaubrand® intraMark, Brand GMBH, Wertheim, Germany), which was subsequently irradiated for each time and then placed inside a thin-wall precision quartz ESR tube with a diameter of 4 mm and a length of 250 mm (Wilmad, Vineland, NJ, USA). Double integration of ESR spectra was performed on WiNEPR processing program (Bruker BioSpin, GmbH). All measurement was recorded under the following conditions: temperature 296 K; microwave frequency 10.03 GHz; microwave power 10 mW; receiver gain 5.0 x 10^4; modulation amplitude 1.00 G; modulation frequency 100 KHz; sweep time 83.89 sec; scan times 10 times.

Singlet Oxygen generation. The 4-oxo-TEMP was used as a spin-trapping reagent for the detection of $^1O_2$ generation by photoexcited C_{60} conjugates (C_{60}-oligo-Lys **1** and C_{60}-PEG **2**). In the presence of $^1O_2$, 4-oxo-TEMP is converted to its $^1O_2$ adduct, 4-oxo-TEMPO, to reveal specific ESR signals. Each aqueous solution of **1** or **2** (0.1 mM in Milli-Q water, 40 µL) was mixed with phosphate buffer (pH 7.0, 300 mM, 20 µL), Milli-Q water (32 µL), and 4-oxo-TEMP (1 M in Milli-Q water, 8 µL) in a 4-mL vial and subjected to oxygen bubbling for 45 sec. In the case of measurements in MeOH-d_4 or chloroform, the solution of **1** or **2** (1 mM in MeOH-d_4 or chloroform, 2 µL) in a 4-mL vial was mixed with 4-oxo-TEMP (1 M in Milli-Q water, 4 µL) and 45 µL MeOH-d_4 or chloroform, and then subjected to oxygen bubbling for 45 sec. An aliquot (35 µL) of each mixed solution was taken and sealed in a glass capillary and subjected to the light irradiation by a green LED. Subsequently, the capillary was placed in an ESR tube for the measurement. After the ESR spectra were taken, obtained signals were analyzed by double integration to quantify the $^1O_2$ generation.

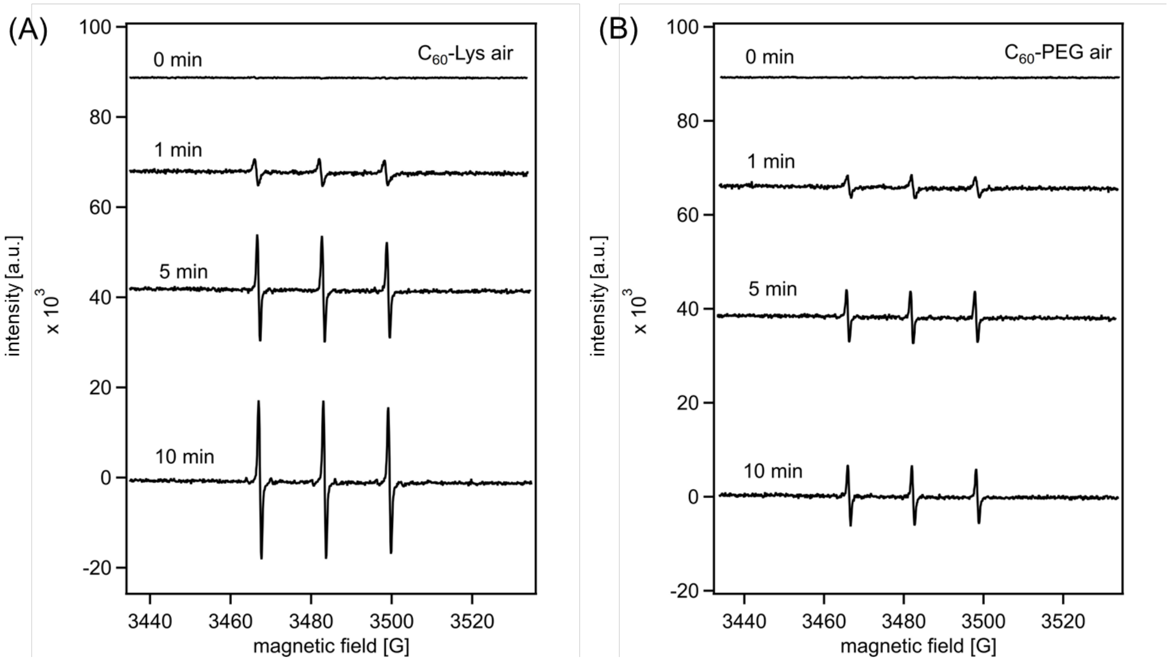

Figure S2. ESR spectra of $^1O_2$ adduct of 4-oxo-TEMP generated in aqueous solutions of C_{60}-oligo-Lys **2** (A) and C_{60}-PEG$_{20}$ **3** (B) under irradiation with a green LED (527 nm) at ambient condition. Final concentration: C_{60}-oligo-Lys **2** or C_{60}-PEG$_{20}$ **3**: 40 μM; 4-oxo-TEMP: 80 mM; phosphate buffer (60 mM, pH 7.0). Irradiation time: 0, 1, 5, and 10 min.

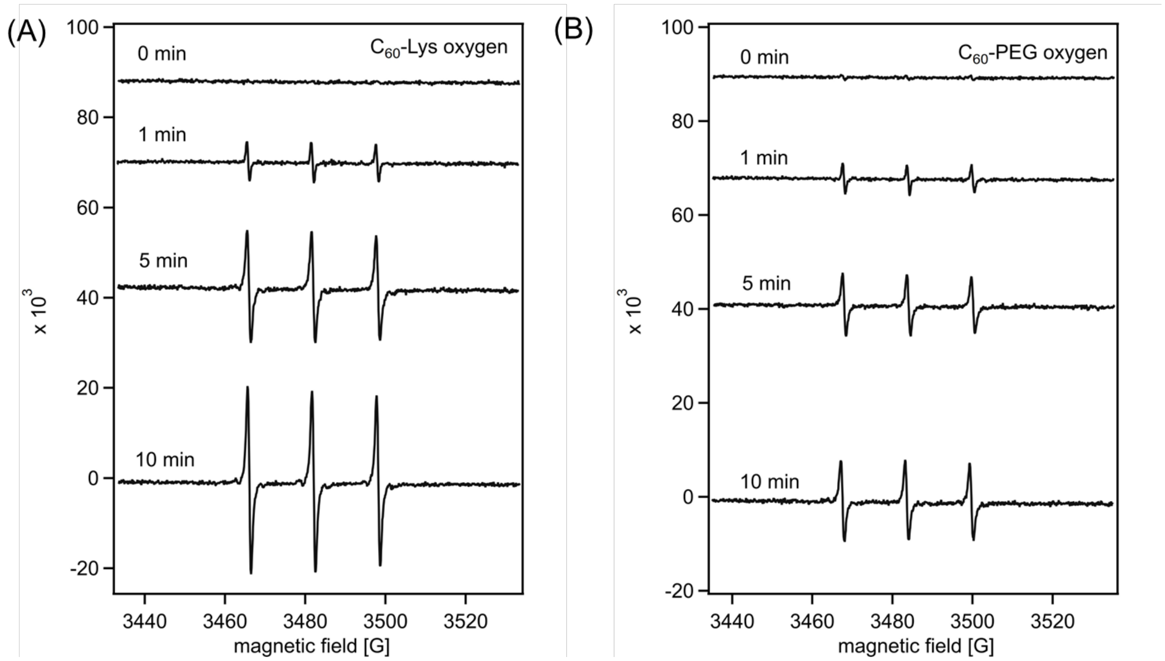

Figure S3. ESR spectra of $^1O_2$ adduct of 4-oxo-TEMP generated in aqueous solutions of C_{60}-oligo-Lys **1** (A) and C_{60}-PEG **2** (B) under irradiation with a green LED (527 nm) at oxygen saturated condition. Final concentration: C_{60}-oligo-Lys **1** or C_{60}-PEG **2**: 40 μM; 4-oxo-TEMP: 80 mM; phosphate buffer (60 mM, pH 7.0). Irradiation time: 0, 1, 5, and 10 min.

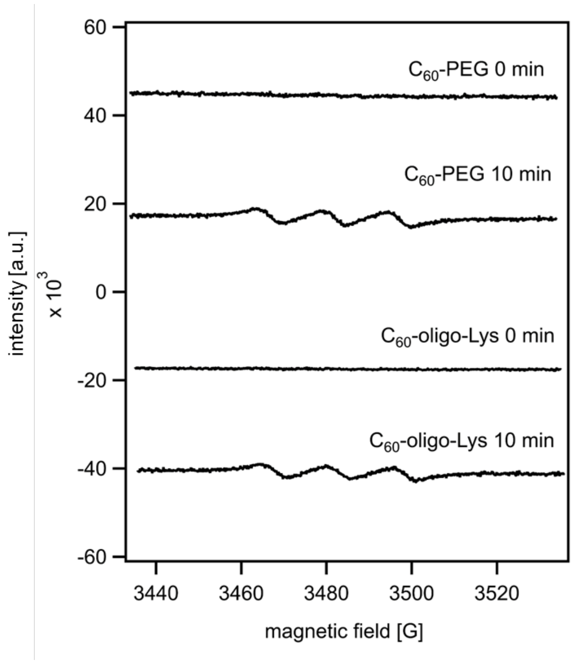

Figure S4. ESR spectra of $^1O_2$ adduct of 4-oxo-TEMP generated in a methanol-d_4 solution of C_{60}-PEG **2** (upper) and C_{60}-oligo-Lys **1** (lower) under irradiation with a green LED (527 nm) at oxygen saturated condition. Final concentration: C_{60}-oligo-Lys **1** or C_{60}-PEG **2**: 40 μM; 4-oxo-TEMP: 80 mM. Irradiation time: 0, 1 and 10 min.

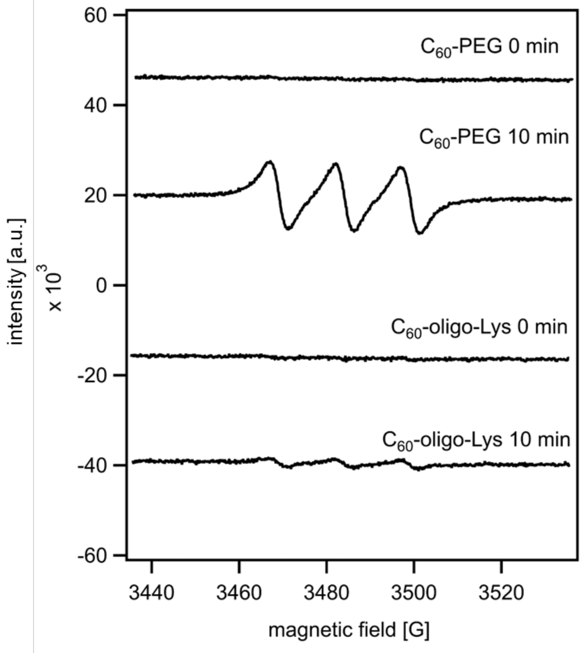

Figure S5. ESR spectra of $^1O_2$ adduct of 4-oxo-TEMP generated in a chloroform solution of C_{60}-PEG **2** (upper) and C_{60}-oligo-Lys **1** (lower) under irradiation with a green LED (527 nm) at oxygen saturated condition. Final concentration: C_{60}-oligo-Lys **1** or C_{60}-PEG **2**: 40 μM; 4-oxo-TEMP: 80 mM. Irradiation time: 0, 1 and 10 min.

$O_2^{\bullet -}$ generation. DEPMPO was used as a spin-trapping reagent to detect photo-induced $O_2^{\bullet -}$ generation by C_{60} conjugates (C_{60}-oligo-Lys **1** and C_{60}-PEG **2**). In the presence of $O_2^{\bullet -}$, DEPMPO is converted to its $O_2^{\bullet -}$ adduct, DEPMPO•OOH, to show specific ESR signals. In a 4-mL vial, each aqueous solution of **1** or **2** in Milli-Q water (0.1 mM, 20 μL) was mixed with DETAPAC (5 mM, 10 μL, metal chelator to avoid subsequent Fenton reaction) in phosphate buffer (pH 7.0, 300 mM), DEPMPO in DMSO (565 mM, 10 μL), NADH in Milli-Q water (100 mM, 5 μL), and L-histidine in Milli-Q water (100 mM, 5 μL, $^1O_2$ quencher) and subjected to oxygen bubbling for 45 sec. In the conditions without L-histidine or NADH, same volume of Milli-Q water was added. An aliquot (35 μL) of each mixed solution was taken and sealed in a glass capillary and subjected to the light irradiation by a green LED. Subsequently, the capillary was placed in an ESR tube for the measurement. After the ESR spectra were taken, obtained signals were analyzed by double integration to quantify the $O_2^{\bullet -}$ generation.

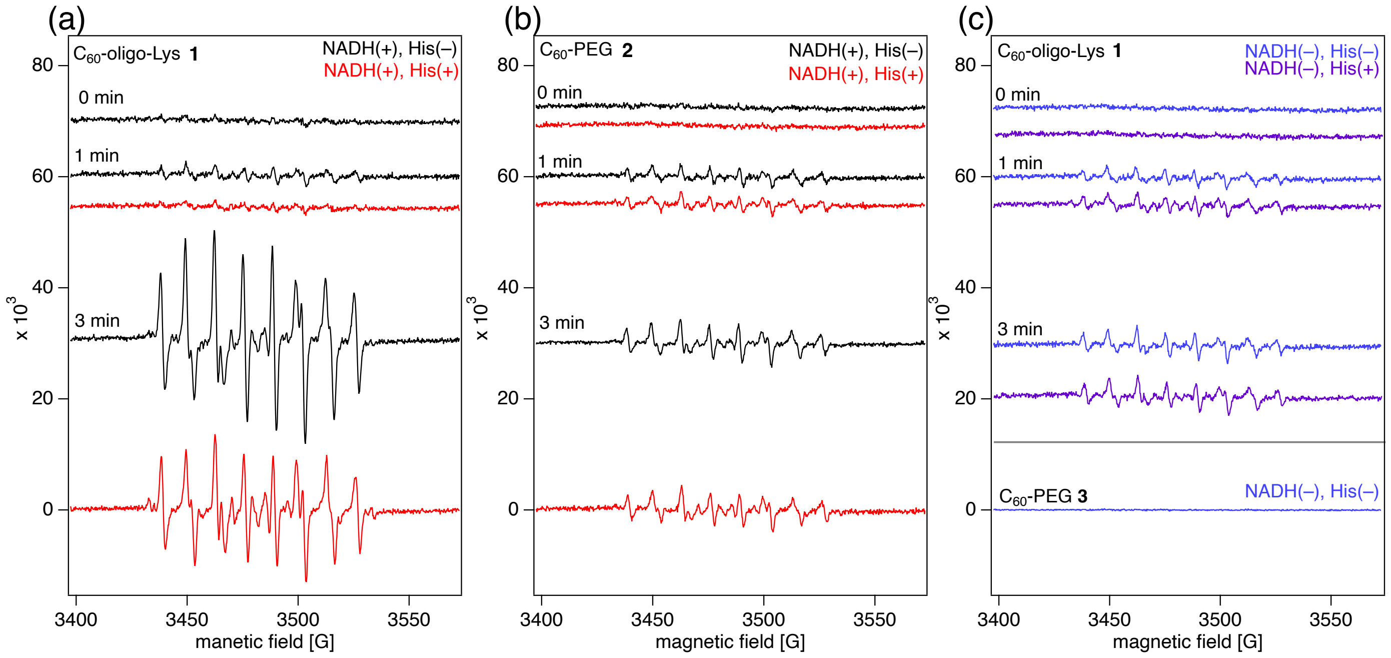

Figure S6. ESR spectra of $O_2^{\bullet -}$ adduct of DEPMPO generated in an aqueous solution of C_{60}-oligo-Lys **1** (a) or C_{60}-PEG **2** (b) under irradiation by a green LED (527 nm) in the presence of NADH and with L-histidine (black), or without L-histidine (red). Or in the absence of NADH (c). C_{60}-oligo-Lys **1** or C_{60}-PEG **2**: 40 μM; DEPMPO: 113 mM; NADH: 10 mM; DETAPAC: 1 mM; L-histidine: 10 mM in phosphate buffer (60 mM, pH 7.0) with 20% DMSO (v/v). Irradiation time: 0, 1, 3 min.

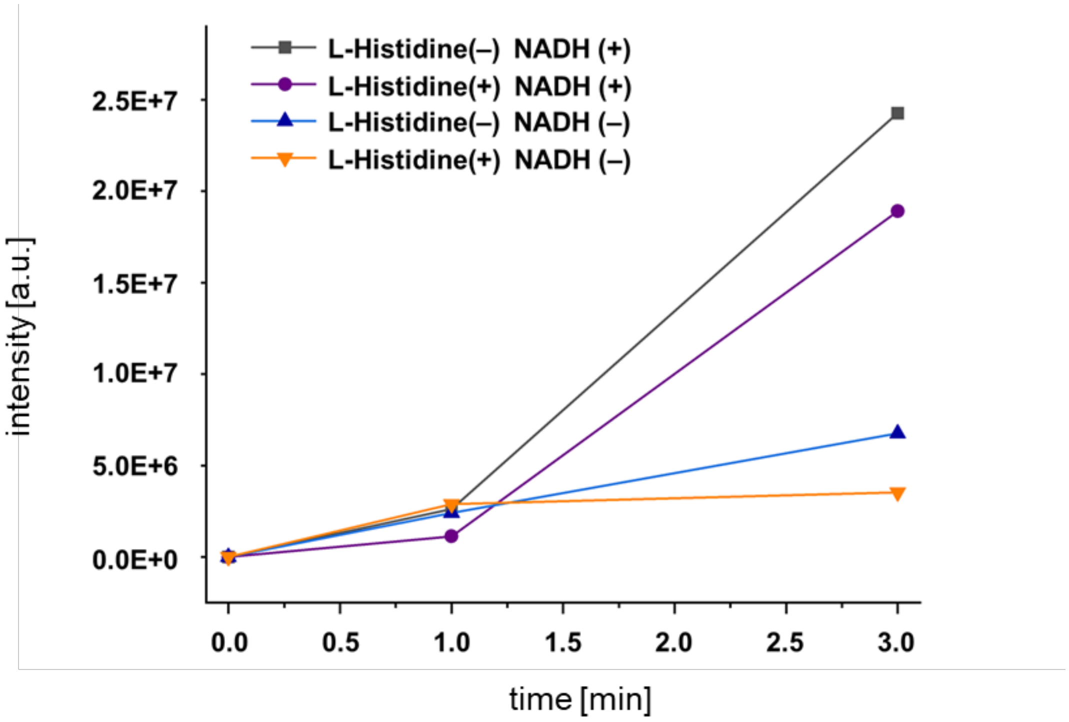

Figure S7. Irradiation time-dependent $O_2^{\bullet-}$ generation by C_{60}-oligo-Lys **1** in the presence/absence of NADH or L-His analyzed by double-integration, detected as $O_2^{\bullet-}$ adduct of DEPMPO. Irradiation: green LED (527 nm).

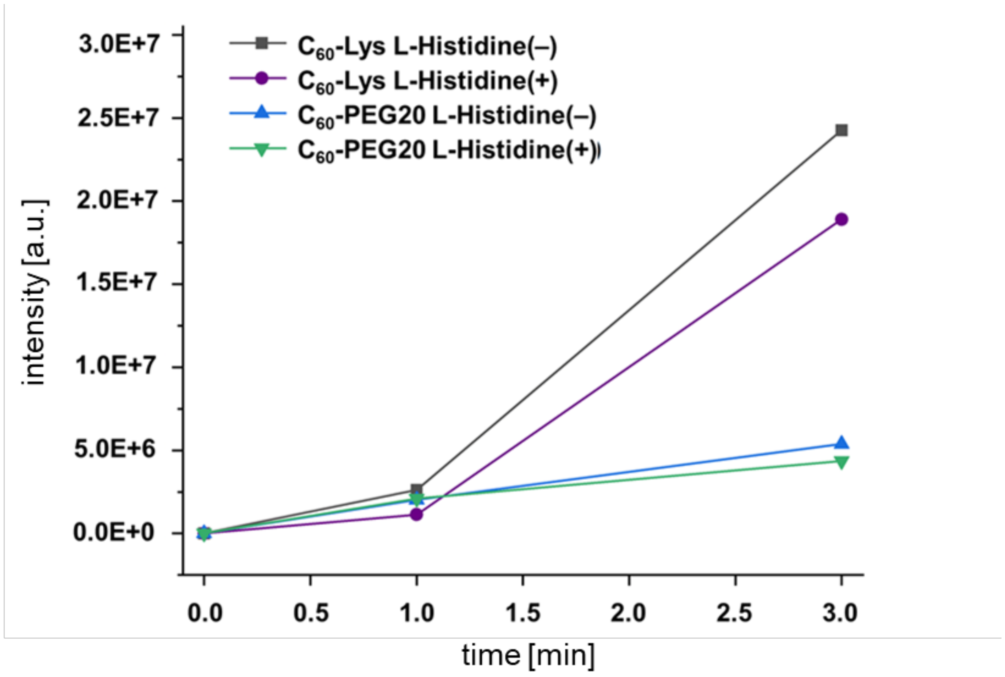

Figure S8. Irradiation time-dependent $O_2{\bullet}^-$ generation by C_{60}-oligo-Lys **1** and C_{60}-PEG **2** in the presence/absence of NADH or L-His analyzed by double-integration, detected as $O_2^{\bullet-}$ adduct of DEPMPO. Irradiation: green LED (527 nm).

Hydroxyl radical generation. DMPO was used as a spin-trapping reagent to detect photo-induced •OH generation by C_{60} conjugates (C_{60}-oligo-Lys **1** and C_{60}-PEG_{20} **2**). In the presence of •OH, DMPO is converted to its •OH adduct, DMPO-OH, to show specific ESR signals. In a 4-mL vial, each aqueous solution of **1** or **2** in Milli-Q water (0.1 mM, 20 μL) was mixed with Fe(II)-DETAPAC (0.2 mM) in 300 mM phosphate buffer (pH 7.0, 10 μL), and DMPO in Milli-Q water (725 mM, 10 μL), and NADH in Milli-Q water (50 mM, 10 μL) and subjected to oxygen bubbling for 45 sec. An aliquot (35 μL) of each mixed solution was taken and sealed in a glass capillary and subjected to the light irradiation by a green LED. Subsequently, the capillary was placed in an ESR tube for the measurement. After the ESR spectra were taken, obtained signals were analyzed by double integration to quantify the •OH generation.

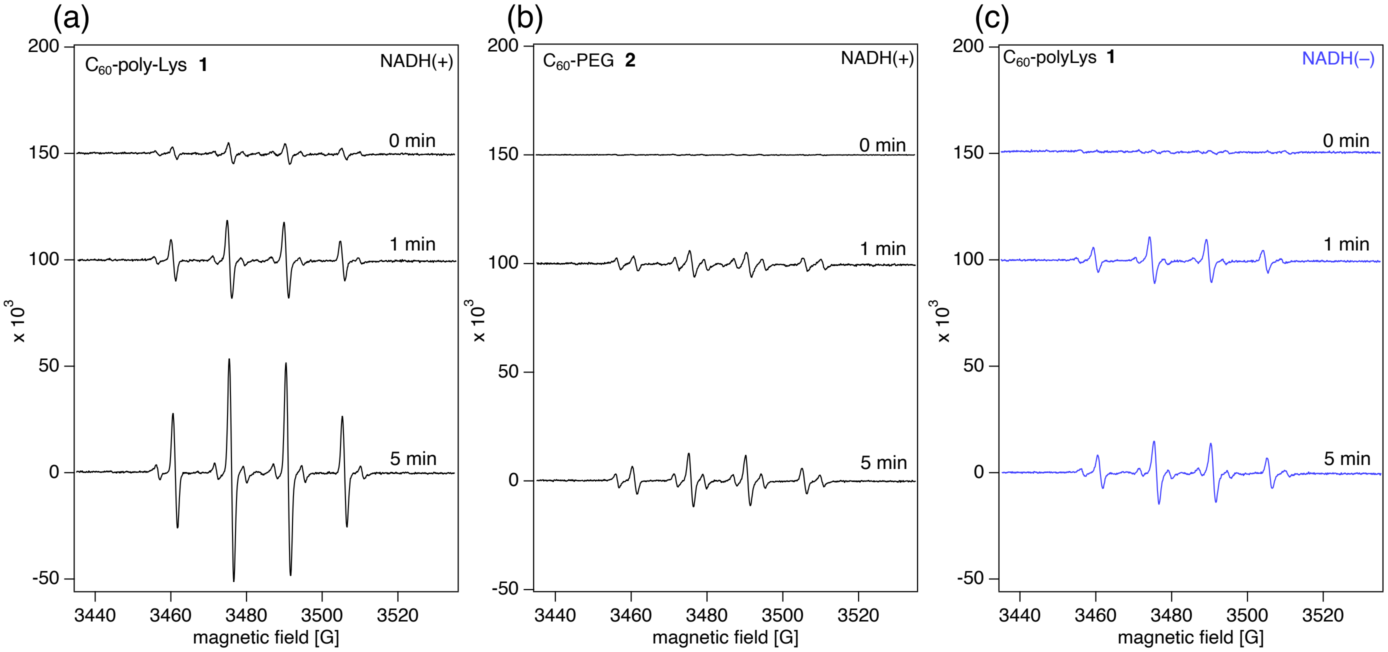

Figure S9. ESR spectra of •OH adduct of DMPO generated in an aqueous solution of C_{60}-oligo-Lys **1** (a) or C_{60}-PEG **2** (b) in the presence of NADH or an aqueous solution of C_{60}-oligo-Lys **1** (c) in the absence of NADH under irradiation by a green LED (527 nm). C_{60}-oligo-Lys **1** or C_{60}-PEG **2**: 40 μM; DMPO 145 mM; Fe(II)-DETAPAC: 40 μM; NADH: 10 mM; in phosphate buffer (60 mM, pH 7.0). Irradiation time: 0, 1, and 5 min.

6. Transient absorption spectroscopy

Pump-probe transient absorption measurements were performed by means of a HELIOS (HE-VIS- NIR) (Ultrafast Systems, Sarasota, FL, USA) femtosecond transient absorption spectrometer by using, as an excitation source, a Solstice-F-1K-230 V laser system (Newport Spectra Physics, Santa Clara, CA, USA), combined with a TOPAS Prime (TPR-TOPAS-F) (Light Conversion, Vilnius, Lithuania) optical parametric amplifier (pulse width: 100 fs, 1 kHz repetition rate, selected output wavelength: 320 nm). The overall

temporal resolution of the system is 300 fs. Air-equilibrated solutions in 0.2 cm optical path cells were analyzed under continuous stirring. The pump energy on the sample was 4 μJ/pulse. Surface Xplorer V4.5 software from Ultrafast Systems was used for the data acquisition and analysis. The 3D data surfaces were corrected for the chirp of the probe pulse prior to the analysis. Lifetimes were taken as average of values derived from the fitting of several decays in selected ranges. Errors on lifetimes were estimated as the errors reported by the fitting software for each lifetime.

7. Photocytotoxicity

HeLa cells were obtained from ATCC (Manassas, VA, USA). MTT was purchased from Sigma-Aldrich Co. (St. Louis, MI, USA). Gibco™ DMEM high glucose, GlutaMAX™ Supplement (with phenol red, cat. No. 11594446) was purchased from Thermo Fisher Scientific Inc. (Waltham, Massachusetts, USA) and used for usual cell culture after adding 10% of Gibco™ Fetal Bovine Serum (cat. No. 11573397, Thermo Fisher Scientific Inc.) Gibco™ DMEM high glucose, no glutamine, no phenol red (without phenol red, cat. No. 11594416) was purchased from Thermo Fisher Scientific Inc. and used in the photoirradiation experiments without adding FBS and penicillin-streptomycin. PBS (–) (pH = 7.4, Mg^{2+}, Ca^{2+} free) and Gibco™ penicillin-streptomycin (10,000 U/mL, cat No 11548876) were purchased from Thermo Fisher Scientific Inc.

The photocytotoxicity tests were performed in 96-well plates (TPP Techno Plastic Products AG, Trasadingen, Switzerland) using Lumidox® II 90-well LED Arrays equipped with the LED lights with a maximum wavelength of 527, 630 or 660 nm (Analytical Sales and Services, Inc., Flanders, NJ, USA). The cell viabilities were tested by the MTT assay method by measuring OD_{560} using an infinite F200PRO plate reader (Tecan Trading AG, Männerdorf, Switzerland). All the cell viability results are means of three independent experiments (n = 3) and are expressed as means ± SE.

Preincubated HeLa cells at their log phase were inoculated in a 96-well plate with a density of ca. $1 \cdot 10^4$ per well and cultured in DMEM (with phenol red) at 37 ºC for 24 h in the atmosphere of 5% CO_2. After the media were removed, 100 μL of C_{60}-oligo-Lys **1** or C_{60}-PEG **2** in DMEM (with phenol red) were added to the cells and incubated in dark at 37 ºC for 24 h in the atmosphere of 5% CO_2. Subsequently, cells were washed with PBS (–) to remove unbound C_{60} derivatives, and DMEM (without phenol red) was added to each well. Cells were treated with light irradiation using by LED for 15 min under the following conditions.

(1) green LED (maximum wavelength at 527 nm) at 25 mW • well^{-1}
(2) red LED (maximum wavelength at 630 nm) at 45 mW• well^{-1}
(3) dark red LED (maximum wavelength at 660 nm) at 65 mW• well^{-1}

After additional incubation at 37 ºC in the atmosphere of 5% CO_2 for 2 h, the medium of each well was replaced with 100 μL MTT assay solution (0.5 mg • mL^{-1} in DMEM without phenol red) and incubated for 2 h. Subsequently, the medium was removed from each well and DMSO (100 μL) was added. The absorbance at 560 nm of the solution in each well was recorded using a plate reader. Each experiment was repeated three times. As a negative control experiments, the cells were treated in the same way but without chemicals.

8. References

1. Y. P. Sun, P. Wang and N. B. Hamilton, *J. Am. Chem. Soc.*, **1993**, *115*, 6378-6381.
2. C. Tanielian, C. Wolff and M. Esch, *J. Phys. Chem.*, **1996**, *100*, 6555-6560.
3. J. Mosinger and Z. Micka, *J. Photochem. Photobiol. A*, **1997**, *107*, 77-82.
